# Recent trends and technological development in plasma as an emerging and promising technology for food biosystems

**DOI:** 10.1016/j.sjbs.2021.12.023

**Published:** 2021-12-16

**Authors:** Catalina J. Hernández-Torres, Yadira K. Reyes-Acosta, Mónica L. Chávez-González, Miriam D. Dávila-Medina, Deepak Kumar Verma, José L. Martínez-Hernández, Rosa I. Narro-Céspedes, Cristóbal N. Aguilar

**Affiliations:** a Bioprocesses and Bioproducts Research Group, Food Research Department, School of Chemistry, Universidad Autónoma de Coahuila, 25280 Saltillo, Coahuila, Mexico; b Agricultural and Food Engineering Department, Indian Institute of Technology Kharagpur, Kharagpur 721 302, West Bengal, India

**Keywords:** Cold plasma, Emerging technology, Food preservation, Food processing

## Abstract

The rising need for wholesome, fresh, safe and “minimally-processed” foods has led to pioneering research activities in the emerging non-thermal technology of food processing*.* Cold plasma is such an innovative and promising technology that offers several potential applications in the food industry. It uses the highly reactive, energetic and charged gas molecules and species to decontaminate the food and package surfaces and preserve the foods without causing thermal damage to the nutritional and quality attributes of food. Cold plasma technology showed promising results about the inactivation of pathogens in the food industry without affecting the food quality. It is highly effective for surface decontamination of fruits and vegetables, but extensive research is required before its commercial utilization. Recent patents are focused on the applications of cold plasma in food processing and preservation. However, further studies are strongly needed to scale up this technology for future commercialization and understand plasma physics for getting better results and expand the applications and benefits. This review summarizes the emerging trends of cold plasma along with its recent applications in the food industry to extend shelf life and improve the quality of food. It also gives an overview of plasma generation and principles including mechanism of action. Further, the patents based on cold plasma technology have also been highlighted comprehensively for the first time.

## 1 Introduction

The number of food-related diseases has increased in recent years, as demand for fresher and safer products has increased (Mandal et al., 2018, Min et al., 2017, Prasad et al., 2017, Ritter et al., 2018). Since consumers demand better food quality, different alternatives have been sought to obtain better food and to satisfy the consumer's demand for food with high nutritional value and safety (Marquez et al., 2017). The demand for products with better characteristics, with a long shelf life and free of microorganisms, has led to the search for treatments in which the product has minimal or no changes and the treatment is also effective against the microorganisms most commonly found in food (Timmons et al., 2018). Damage to food by pathogenic microbes causes widespread economic losses. Some of the most often detected harmful bacteria in food cause disease. In some cases, *Escherichia coli*, *Listeria monocytogenes* and *Salmonella* spp., are the main microbes that cause death in humans (Kim and Min, 2017, Min et al., 2016, Timmons et al., 2018). Various strategies, such as chlorine treatment, ascorbic acid and citric acid, have been used for food decontamination but are less effective as they reduce the small number of pathogens (Min et al., 2016, Prasad et al., 2017). Thermal processing is the most widely used approach for food preservation to control pathogens and food spoilage-provoking microorganisms. Even though heat treatment has numerous drawbacks, such as changes in appearance, textural damage, changes in taste and sensory qualities, and reduction of nutritional characteristics, these are essential factors for the consumer (Mandal et al., 2018, Prasad et al., 2017, Srivastav et al., 2020). Food contamination occurs during the various processes that food is exposed to before it reaches the consumer. Therefore, different methods of decontamination that do not cause food damage have been reported in recent years, they are as follows: cold plasma (CP), dielectric heating (radiofrequency and microwave heating), high-pressure processing (HPP), infrared (IR), ohmic heating, ozone processing, pulsed electric field (PEF), pulsed light (PL), ultrasound, etc. (Al-Hilphy et al., 2016, Chizoba Ekezie et al., 2017a, Chizoba Ekezie et al., 2017b, Srivastav et al., 2020, Verma et al., 2020a, Verma et al., 2020b).

Cold plasma has recently developed as a novel technique for assessing chemical and microbial hazards in food (Min et al., 2016). Cold plasma is not only a low-pressure system. It can also be an atmospheric pressure system. This system is used in various industries such as automotive, electronics, medical, textile, household appliances and materials. It has also been applied to biotechnology, nanotechnology, environmental technology and others (Bahrami et al., 2016, Cahill et al., 2017, Claro et al., 2015, Karami-Gadallo et al., 2017, Khadtare et al., 2017, Kovalova et al., 2016, Metelmann et al., 2018, O’Connor et al., 2014, Olschewski, 2011, Yagub et al., 2014). Recently, it has gained a great deal of interest in the food industry because of the advantages following its application. Cold Plasma has been used to inactivate microorganisms in various food and food products, such as apples (Tappi et al., 2019), lettuce (Min et al., 2017), carrots (Schnabel et al., 2015), tomatoes (Min et al., 2018), blueberries (Lacombe et al., 2015), eggshells (Dasan et al., 2018), black pepper (Mošovská et al., 2018), almonds (Hertwig et al., 2017a), meat (Misra and Jo, 2017), fish products (Albertos et al., 2017) and ready to eat ham (Yadav et al., 2019). It has been used in the food industry to inactivate microorganisms most commonly found in food, such as *E. coli* (Segura-ponce et al., 2018), *Salmonella* (Timmons et al., 2018) and *L. monocytogenes* (Bauer et al., 2017). In addition, aflatoxigenic spores of certain microbes have also been used for inactivation. For example, aflatoxigenic spores of *Aspergillus flavus* and *A. parasiticus* in hazelnuts (Dasan et al., 2017).

**Fig. 1:**
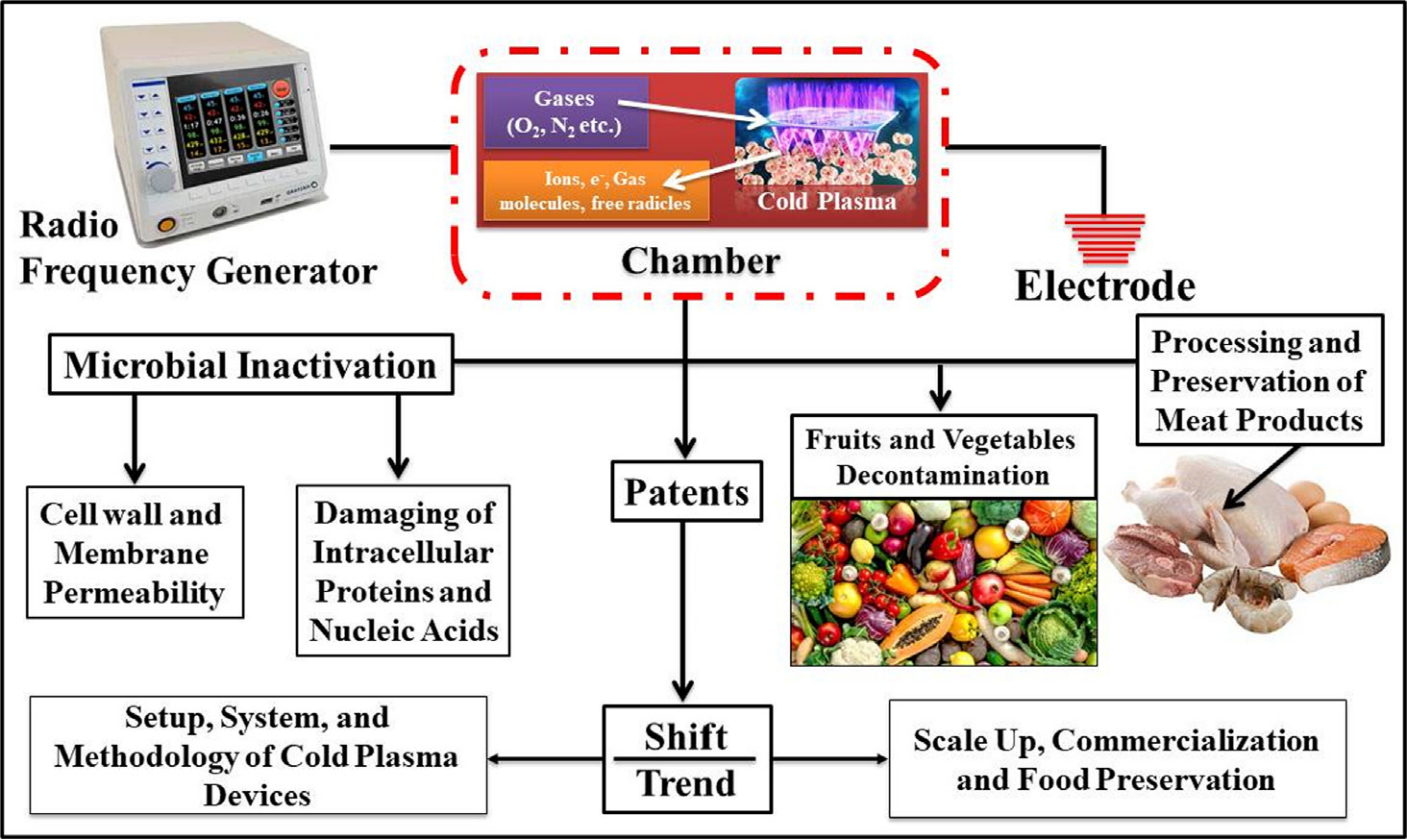
A graphical summary of this study of recent trends and technological developments in plasma as an emerging and promising technology for food biosystems.

Although, there is a lot of literature and published reports in this area. In this context, positive results have been observed in the inhibition of microorganisms and the conservation of food. Therefore, the subject of cold plasma continues to be novel for its application in different areas of food for different purposes because it has several advantages such as water-saving, low energy use during the process, maintenance of the characteristics and nutrients of food after treatment. The objective of this review is to comprehensively discuss the recent findings to highlight the important plasma aspects and emerging developments in the food processing and preservation arena. The review also covers the mechanism for plasma action and its effect on microorganisms. In addition, this review also highlights the existing patents as well as the technological developments in plasma technology, with a discussion on future potentials and challenges. The present study would promote early acceptance of this eco-friendly technology by the food processing sector and regulatory authorities; so that the fullest worth of its commercial applications will be explored. Fig. 1 accurately depicts the overview of this review study.

## 2 Brief overview on plasma: Perspective and trends

Plasma is the fourth state of matter, an ionized gases mixture, composed primarily of photons, ions, and electrons as neutral atoms with a simultaneous electric interaction between particles and atoms in its excited state (Olschewski, 2011, Khamsen et al., 2016a, Khamsen et al., 2016b, Pankaj and Keener, 2017, Park and Ha, 2018). It is commonly seen as bright fluorescent light; Langmuir, 1928, coined the word “plasma” after seeing oscillations in ionized gas (Langmuir, 1928) and describing it as an “area having equilibrium charges of ions and electrons.” Plasma status can naturally be found in aurora borealis, stars, fluorescent or neon gaslight, etc., it is commonly known as light fluorescence (Mandal et al., 2018).

Plasma technology has a variety of applications, such as medical (Cahill et al., 2017, Kovalova et al., 2016, Lehmann et al., 2017, O’Connor et al., 2014), environmental technology (Mustafa et al., 2018), polymer modification (Alam et al., 2017, Khamsen et al., 2016b), nanotechnology (Khadtare et al., 2017), textile industry (Zhang et al., 2017a) and biotechnology (Albertos et al., 2017, Tappi et al., 2019, Ulbin-Figlewicz et al., 2013). Recently it has been used for the food industry obtaining positive results for the inhibition of microorganisms, enzyme inactivation, food preservation, food packaging modification (Table 1) (Bahrami et al., 2016, Chizoba Ekezie et al., 2017b, Kumar et al., 2017, Sarangapani et al., 2018).

**Table 1:** Summary of recent findings on the influence of cold plasma on the quality attributes of food products and food packaging materials.

Products	Plasma generating Source	Processing Parameters or Plasma Source							Major Findings	References
		Frequency	Power (W)	Time (min)	Gas	Voltage	Flow Rate	Pressure		
***Microorganisms Inhibition***										
Korean Rice Cakes	Dielectric barrier discharge (DBD)	60 Hz	–	1–5	–	26 kV	–	–	*Salmonella* growth is reduced by 3.9 ± 0.3 log CFU/g.	Kang et al. (2021)
Fresh cut carrot	DBD	60 Hz	–	1–5	*	60–80 kV	–	–	Mesophiles as well as the yeasts and mold count reduced by 2.1 log_10_ CFU/g.	Mahnot et al. (2020)
Chicken	DBD	60 Hz	233	1–5	Oxygen and nitrogen	100 kV	–	–	Mesophiles, psychrotrophs and Enterobacteriaceae count were reduced by 1.5, 1.4 and 0.5 log.	Moutiq et al. (2020)
Fresh strawberries and spinach	DBD	–	900	5–27	–	100 kV	–	–	*E. coli* reduced by 2–2.2 log_10_ CFU/ml and 1.3 and *L. innocua* reduced by 1.3–1.7 log_10_ CFU/ml, respectively.	Ziuzina et al. (2020)
Grape tomatoes, apples, cantaloupe and romaine lettuce	–	–	–	0.17–1	–	17 kV	–	7 psi	*Listeria* growth reduced by 5 log CFU/piece.	Song & Fan (2020)
Blueberry	DBD	–	–	0.83	–	45 kV	–	–	Inhibited the growth of B. cinerea	Zhou et al. (2019)

***Enzyme Inactivation***										
Açai pulp	DBD	50–750 Hz	–	5–15	–	20 kV	–	1 bar	Polyphenol oxidases (PPO) and peroxidases (POD) activities were reduced after treatment.	Dantas et al. (2021)
Whole bananas	DBD	10 kHz	–	1–2	–	40 V	–	–	PPO) and POD activities were reduced after treatment.	Gu et al. (2021)
Banana Peel	DBD	13.5 kHz	200	1–30	–	–	–	6 bars	PPO activity was reduced to 46% of the original activity.	Wohlt et al. (2021)
Fresh-cut pears	DBD	–	–	1–5	–	45–65 kV	–	1 bar	After treatment, peroxidase and pectin methylesterase (PME) activities were reduced.	Zhang et al. (2021)
Camu-camu juice	–	200–960 Hz	–	15	–	24 kV	–	–	Cold plasma reduced the activity of PPO and POD.	de Castro et al. (2020)
Green coconut water	DBD	200–730 Hz	–	15	–	15–20 kV	–	1 bar	Complete inactivation of POD was achieved.	Porto et al. (2020)

***Food Preservation***										
Blue swimming crab (*Portunus armatus*)	DBD	50 Hz	–	0–15	–	80 kVRMS	–	1 bar	Refrigerated storage life was increased to 12 days.	Olatunde et al. (2021a)
*Litopenaeus vannamei*	DBD	50 Hz	–	10	Argon and atmospheric air	16 kVRMS		1 bar	PUFA and protein oxidation were reduced and shelf life was extended to 18 days.	Shiekh et al. (2021)
Chicken	DBD	60 Hz	233	1–5	Oxygen and nitrogen	100 kV	–	–	The storage period was extended up to 24 days due to microbial inactivation.	Moutiq et al. (2020)
Strawberries	DBD	50 Hz	–	10–30	–	60 kV	–	1 bar	The microbial load was reduced and refrigerated shelf life was increased to 9 days.	Rana et al. (2020)
Fresh strawberries and spinach	DBD	–	900	5–27	–	100 kV	–	–	*E. coli* reduced by 2–2.2 log_10_ CFU/ml and 1.3 and *L. innocua* reduced by 1.3–1.7 log_10_ CFU/ml, respectively.	Ziuzina et al. (2020)
Asian sea bass slices (*Lates calcalifer*	DBD	50 Hz	–	5	Argon and oxygen	16 kVRMS	–	1 bar	Lipid oxidation reduced and shelf life increased to 15 days.	Singh & Benjakul (2020)

***Food Packaging***										
Phlorotannin (PT)/*Momordica charantia* polysaccharide (MCP) packaging film	–	–	350	0.5	Nitrogen	–	100 cm^3^/min	–	The release of PT was enhanced and the antimicrobial activity of packaging film increased.	Cui et al. (2020)
Whey and gluten protein-based edible films	Glow discharge (GD)	20 kHz	50	5–15	–	–	–	–	Gas permeability of edible film was decreased and tensile strength was increased.	Moosavi et al. (2020)
Bi-layer protein films	GD	60 Hz	5–11	1–5	–	4.4 kV	–	10 Pa	Tensile strength increased by 175% and water vapor permeability was decreased by 65%.	Romani et al. (2020)
Casein edible films	DBD	–	–	0.25–2	–	30–70 V	–	–	Packaging parameters notably tensile strength, elongation, thermostability, and barrier characteristics were enhanced.	Wu et al. (2020)
Cassava starch films	DBD	50 Hz	–	1–20	–	31 kV	–	–	The water vapor barrier and mechanical strength of the film were significantly higher.	Heidemann et al. (2019)
Starch-based films		13.56 MHz	70	30	–	–	0.35 cm^3^/min	0.4-0.045 mbar	The hydrophobic property of the film was improved.	Sifuentes-Nieves et al. (2019)

Plasma technology can be categorized according to generation, thermal and low-temperature plasma methods. Different authors report that thermal plasma consists of thermodynamically balanced ions, electrons and gas molecules at temperatures of approximately 20,000 K. In all plasma components, the temperature of the gas is almost the same and very high (4–20 × 103 °C) (Liao et al., 2017a, Mandal et al., 2018). Low-temperature plasma is usually divided into quasi-equilibrium plasma (100–150 °C) where there is a local thermodynamic equilibrium between species such as electrons and gas molecules and non-equilibrium plasma (<60 °C) where electrons have higher temperatures and gas one molecule has moderate temperatures without any local thermodynamic balance, resulting in lower temperatures for the entire system. Various authors refer to non-equilibrium plasma under various names such as Simply Cold Plasma (CP), Atmospheric Cold Plasma (ACP), or Non-thermal Plasma (NTP) (Mishra et al., 2016, Stoica et al., 2014). Cold plasma can be produced by a radiofrequency generator or by atmospheric pressure (Coutinho et al., 2018, Thirumdas et al., 2014).

**Fig. 2:**
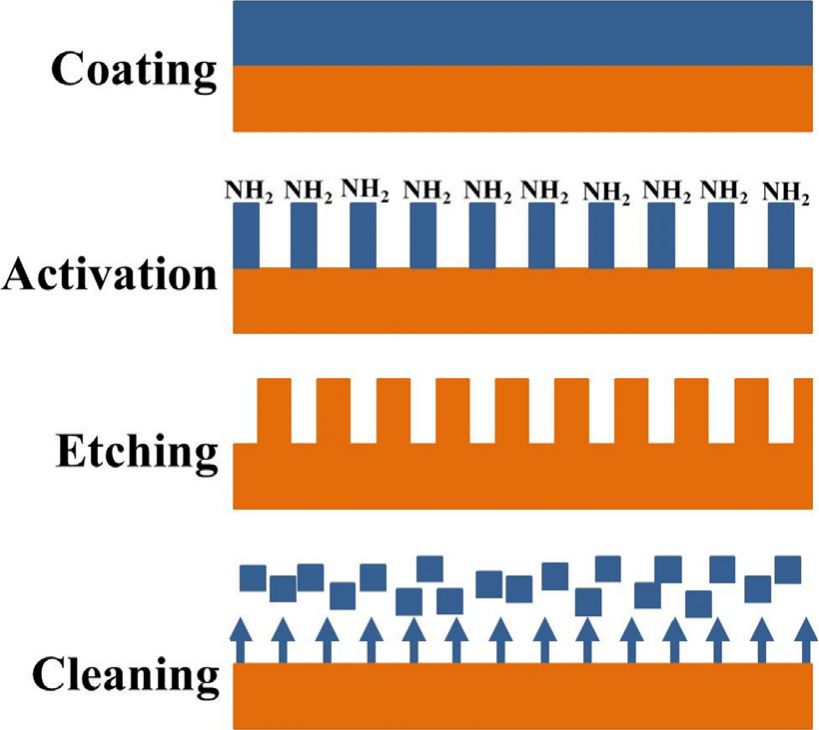
Processes performed during plasma treatment.

The plasma equipment consists of a vacuum chamber (in some cases), a radiofrequency generator, an electrode, and a control unit. Cold plasma can be produced in gases by electrical discharges with less power input in atmospheric or vacuum pressure (Ritter et al., 2018). To reduce the pressure inside the plasma vacuum chamber, it is reduced to approximately 0.1–0.5 mbar. The process gas is introduced into the chamber for the formulation of the plasma, which depends on the treatment to be used and the substrate material. Plasma is used for cleaning, activating, etching and coating as shown in Fig. 2 (Prasad et al., 2017, Rothrock et al., 2017). Reactive gases, such as oxygen and inert gases, such as N_2_, have a more aggressive effect on the surfaces to be modified than inert gases so that the former is mainly used for etching and the latter to clean and activate the surfaces. On the other hand, monomeric gasses or liquids, the former, such as ethylene and propylene, and the latter, which have to pass into the gaseous state due to the vacuum produced by the vacuum pump, and then transformed into the plasma to produce a plasma polymerization reaction on the surface of the sample or material to be modified, may also be used to produce a polymerization reaction. They are mainly used for coating; however, if they are used at very short times, the surface can only be activated.

## 3 Application and mechanisms of action of plasma

Cold plasma has been used in recent years for the inactivation of microorganisms in food such as pathogenic bacteria, while plasma was initially patented in 1968 as a sterilization method (Menashi, 1968, Bauer et al., 2017, Thomas-Popo et al., 2019). Mechanisms responsible for cold-plasma inactivation of microbial organisms have not been fully described, mainly various plasma growth methods, processing conditions, device characteristics, variations in the microbial properties and sensitivity, etc. However, different authors have reported that the process of damaging bacteria occurs in three different mechanisms (Ritter et al., 2018). One of the first mechanisms reported is the cell membrane and wall permeability (pore formation, permeability, and disruption), which leads to the leakage of cellular components such as potassium, nucleic acids, and proteins; the second mechanism is damage to intracellular proteins, and the last mechanism reported is damage to chemical nucleic acids (Coutinho et al., 2018, Ritter et al., 2018). Inactivation mechanisms may vary between microorganisms, bacterial spores that are more resistant than bacteria, fungi, and viruses (Coutinho et al., 2018, Hertwig et al., 2015, Stoica et al., 2014).

During plasma treatment, different plasma components and chemical elements such as ozone (O_3_), oxygen (O_2_), nitrogen (N_2_), hydroxyl (OH), energy and free radicals are generated. These components are coupled with highly energetic electrons found at high temperatures and speeds, and different authors report that these electrons are responsible for causing cell damage to the surface of the cell wall of microorganisms, which means that the combination of these components does not allow pathogens to generate resistance to these factors. When microorganisms are exposed to plasma treatment, radicals such as ^•^OH and ^•^NO which are absorbed from the surface of the bacteria and form volatile compounds (CO_2_ and H_2_O) may cause damage to the surface of the cells that are irreparable, resulting in cell death (Misra and Jo, 2017). Hydroxyl radicals (^•^OH) in plasma have toxic effects due to an increase in the permeability of the membrane that compromises the lipids and causes protein damage. At this point, the reactive protein species react with the amino acid chains and cause changes in protein structure, damage to cells, spores, and damage to nucleic acids (Stoica et al., 2014). As a result, energetic electrons, atomic and molecular radicals, and excited molecules can induce microbial etching, allowing bacteria to penetrate and inflict lesions on the surface of the microorganism. However, the microbial response also depends on the bacterial growth mode, with bacteria growing in biofilms requiring a longer exposure time before they become inactivated (Afshari and Hosseini, 2014, Hertwig et al., 2017b, Lacombe et al., 2017, Stoica et al., 2014).

### 3.1 Plasma effect in microorganisms

Gram-positive bacteria are generally more resistant to cold plasma treatment than Gram-negative bacteria due to the thinner membrane structure (Ermolaeva et al., 2011, Fernández et al., 2013, Klämpfl et al., 2012). Authors like Ziuzina and coworkers have reported that both *Salmonella* and *E. coli* Gram-negative bacteria have been more rapidly inactivated in tomatoes than Gram-positive bacteria *L. monocytogenes* (Ziuzina et al., 2014). The thicker membrane of Gram-positive bacteria may present a barrier to the diffusion of reactive plasma species through the bacterial cell wall, thus affecting antimicrobial efficacy.

Cold plasma has not only been used to inactivate bacteria but has also been used to investigate the effects of cold plasma treatment on aflatoxigenic spores of *A. parasiticus* and *A. flavus* in hazelnuts (Dasan et al., 2017). Hazelnuts were infected with *A. parasiticus* and *A. flavus* in this study and then treated with plasma nitrogen or dry air for up to 5 min. With the reference voltage and frequency used, the decontamination effect on the spores of *Aspergillus* spp. was increased. Fungicidal effects with 4.09 log and 4.17 log on *A. parasiticus* and *A. flavus* respectively were observed after 5 min of air plasma treatment. Oxygen plasma was found more advantageous than nitrogen plasma in the decontamination of *Aspergillus* spp. spores (Dasan et al., 2017). The authors claim that this is because oxygen plasma is more reactive and therefore more aggressive to these fungi than nitrogen plasma which is considered to be inert gas plasma. During the plasma process, aflatoxigenic spores remained on hazelnuts, but, since they could not continue to grow at 25 °C for 30 days during storage, thus, they were considered to be damaged cells. The damage caused by *Aspergillus* spp. spore cells were seen by the application of scan electron microscopy (SEM) (Dasan et al., 2017). Recently, Thomas-Popo et al. (2019) used cold atmospheric plasma to decontaminate wheat grain, generally used to produce different types of flour, inactivating Shiga-toxin-producing *E. coli, S. enterica,* and natural microflora. In their study, portions (10 g) of grains inoculated with a 5-strain mixture of *E. coli* or *S. enterica* were used to obtain an initial count of ∼7.0 log_10_ CFU/g. Inoculated or non-inoculated wheat grains were sealed in plastic bags filled with atmospheric air and exposed to ACP at a potential of 44 kV at different times (5, 10, 15 and 20 min) and the pathogen survivors were evaluated after 48 h of incubation (35 °C). Thomas-Popo et al. (2019) demonstrated the inactivation of Shiga-toxin-producing *E. coli* and *S. enterica* after 20 min of treatment with ACP, thus showing the treatment capacity to destroy the survival pathogen. These authors also concluded that flour produced from ACP treated wheat grains may be of high microbial quality.

Cold plasma is a recent technology for the food industry. Positive results can be achieved from this technology, such as the deactivation of microorganisms at low temperatures; it is easy to reproduce, short in time, does not use water or solvents and can meet the required environmental standards, like many other technologies. However, it shows poor results in the case of laboratory comparison, which must be addressed by scientists. Another feature is that due to the size, volume and roughness of the food, the rough surface of some products provides numerous sites for microorganisms to adhere to and potentially escape antimicrobial treatment. It is worth mentioning that the surface that is not in contact with the plasma can not be modified, which is why several studies have worked with agitation during the plasma process to ensure that the treatment is consistent across all surfaces (Coutinho et al., 2018, Misra et al., 2011).

Pathogenic microorganisms are often linked to diseases caused by food intake. Efficient technologies for the deactivation of various pathogenic microorganisms in food have been sought over the last several years, but the physical, nutritional, or organoleptic properties of food must not be damaged by these effective technologies. As we all know, worldwide illnesses caused by contaminated food have been reported to have caused consumers to demand higher food quality. Various microorganisms, such as fungi, bacteria, etc., have been reported to be responsible for foodborne diseases, including *Vibrio cholerae, Staphylococcus aureus, enteric Salmonella, E. coli, L. monocytogenes* and others (Dasan and Boyaci, 2017, Kim and Min, 2017, Min et al., 2016, Prasad et al., 2017, Timmons et al., 2018). Although sterilization is often used to deactivate pathogenic microorganisms that affect food, many authors have recently used cold plasma as an alternative to sterilization for many microorganisms (Cui et al., 2018, Dasan and Boyaci, 2017, Ma et al., 2017, Prasad et al., 2017, Song et al., 2015), especially for *L. monocytogenes* (Kim et al., 2015, Segura-Ponce et al., 2018), *Salmonella* (Georgescu et al., 2017, Kim and Min, 2017, Kim and Min, 2018, Timmons et al., 2018, Won et al., 2017, Yong et al., 2015b). Timmons et al. (2018) used surface dielectric discharges that are Atmospheric Cold Plasma to inactivate pathogens in walnut shells and cherry tomatoes inoculated with pathogens such as enteric *Salmonella, E. coli, and L. monocytogenes*. During the different types of treatments, the inactivation of the different pathogens was observed. Physical cellular damage was shown by scanning transmission microscopy 2–4 min after cold plasma treatment.

### 3.2 Plasma applied to food processing and preservation

#### 3.2.1 In fruits and vegetables decontamination

The consumer’s demand for fresher fruit and vegetables has led to the search for different alternatives to extend shelf life, although packaging with modified atmospheres, low-temperature storage and dehydration are among the most commonly used and sometimes insufficient to extend the shelf life of fruit and vegetables to maintain their high quality (Marquez et al., 2017, Pasquali et al., 2016). After harvesting, fruits and vegetables undergo different changes, such as dehydration, wrinkles, color changes, etc. As a result of these changes, the product is no longer satisfying to the consumer and therefore leads to significant economic losses (Ayhan, 2017). Good manufacturing practices (GMP) and packaging materials help to reduce the different changes that fruit and vegetables undergo during their shelf life. However, these parameters are not sufficient to stop their deterioration due to the different pathogens responsible for the damage to fruit and vegetables. Today's primary challenge is to find effective treatments and technologies for various pathogens without causing sensory or physical alterations to fruits and vegetables (Ayhan, 2017, Dinika et al., 2020).

Most of the minimally processed foods are decontaminated by washing them with chlorine (50–200 mg/L). However, consideration is being given to the formation of carcinogenic compounds due to chlorine, and new alternatives have been sought for their decontamination (Pasquali et al., 2016). Other decontaminants used are ascorbic and citric acids. However, the food is often affected by pH and sensory parameters, as the fruit and vegetables contain approximately 80% moisture (Ramos et al., 2013). In this way, it becomes difficult to maintain food storage conditions at low temperatures because they affect water loss and lead to poor quality stability (Bußler et al., 2017).

Various technologies have been used to extend the shelf life of fruit and vegetables, such as modified atmospheric packaging, coatings, film, preservatives, irradiation and radiation (Kumari et al., 2017). Moreover, various materials have also been used, such as waxes, polysaccharides, casein, proteins, gelatin, gluten, chitosan, aloe vera, etc. These materials have been used individually or in combination, but have not been sufficient to produce a positive result in the expected results. Knowledge on this subject needs to be broadened because not all research meets the standards required by the consumer (Fai et al., 2016, Ganiari et al., 2017, Kumari et al., 2017, Soradech et al., 2017). In the food industry, the use of cold plasma in food and food products based on fruit and vegetables to enhance shelf life has attracted considerable attention. It has been used for the sterilization and cleaning of fruit and vegetables without altering their appearance and nutritional quality, such as melons (Tappi et al., 2016), kiwi (Ramazzina et al., 2015), apples (Bußler et al., 2017), cherry tomatoes (Misra et al., 2014), blueberry (Sarangapani et al., 2016), lettuce leaves (Min et al., 2017), chicory leaves (Pasquali et al., 2016) and others. The temperature of the cold plasma is almost equal to the ambient temperature, which is why this technique is suitable for processes involving thermosensitive food. Although the scope of its potential use in food packaging has also been increased. The efficacy of plasma treatment has been tested for various food products and microbial species (Athukorala et al., 2009, Min et al., 2016, 2017). The efficacy of the plasma is also affected by the many factors that is utilized, such as the kind of substrate and the characteristics of the microorganism to be deactivated.

Plasma ions can catalyze oxidation processes inside and outside the cell, resulting in a decrease in oxidation, depending on the type of gas used and what you want to do on the surface. Oxidation may be higher in some cases (Bußler et al., 2017). Table 2 shows the conditions of cold plasma treatment reported in recent studies aimed at preserving and extending the shelf life of fruit and vegetables free of microorganisms. Table 2 also shows that atmospheric cold plasma was the most widely used in previous works. The use of Oxygen and Nitrogen (30/70) could also be done in this type of process. Tappi et al. (2019) conducted a study in which some quality parameters were measured in apple slices belonging to 4 different types of crops, such as Pink Lady, Fuji, Red Delicious, and Modi, resulting in a reduction of 80% in surface oxidation in all crops, when apple slices were treated with atmospheric plasma. In addition, in another previous report, Ramazzina et al. (2015) assessed the atmospheric effect of cold plasma on the quality of freshly cut kiwi fruit, the treatment consisted of 10 and 20 min per side, four days after storage, and visual quality parameters, texture; chlorophyll, carotenoids and polyphenols were evaluated. After storage time, it was concluded that plasma treatment improved color retention, reduced brown color during storage, no changes in texture were observed compared to control, and also no changes in antioxidant and antioxidant activity were observed.

**Table 2:** Recent findings summarizing the use of cold plasma in food products based on different fruits and vegetables.

Food and Food Products	Plasma generating Source	Processing Parameters or Plasma Source							Microorganism	Major Findings and Remarks	Reference
		Frequency	Power	Time	Gas	Voltage	Flow Rate	Pressure			
Fresh Strawberries and Spinach	Atmospheric cold plasma	–	900 W	5, 7, 10, 13, 20, 22, 24 and 27 min	Ozone	0–100 kV	–	–	*E. coli* and *L. innocua*	Continuous treatment was effective against *L. innocua* inoculated on strawberries, with 3.8 log10 CFU/ml reductions achieved	Ziuzina et al. (2020)
Blueberry Juice Quality	Cold Plasma Jet	1000 Hz	–	2, 4 and 6 min	Argon (Ar) and Oxygen (O_2_)	11 kV	1.0 L/min	–	*Bacillus* spp.	The increment of treatment time and O_2_ concentration significantly promoted an increasing trend of death for *Bacillus*. Compared with thermal treatment, the content of phenolics was significantly increased by CP treatment, and also CP treatment could better keep the original color of blueberry juice	Hou et al. (2019)
Fresh Cut Apple	Cold Plasma	–	29.6 W	3, 5, 10, 15, and 20 min	Nitrogen (N_2_), Ar, O_2_, and Ar-O_2_	–	40 ml/min	1300 to 1370 mTorr for N_2_, 850 to 920 mTorr for Ar, 1300 to 1340 mTorr for O_2_, and 950 to 1000 mTorr for Ar-O_2_ mixture	*Escherichia coli and Listeria innocua*	The treatments using Ar, O_2_, or Ar-O_2_ mixture for 20 min were the most effective to inactivate *E. coli* with O_2_, while the treatment with N_2_ for 20 min reduced *L. innocua* the most for (p < 0.05).	Segura-ponce et al. (2018)
Tomato	Atmospheric Cold Plasma	50 Hz	–	5, 10, 15, and 30 min	–	15 and 60 kV	–	–	*E. coli*	The highest log reduction of 6 log CFU mL^−1^ was achieved in a population of E. coli after 15 min of ACP treatment at 60 kV, which was sustained up to a storage duration of 48 h	Prasad et al. (2017)
Apple slice of different types (Pink Lady, Fuji, Red Delicious, Modi)	Cold Plasma	–	150 W	30 and 60 min	–	150 W	–	–	–	A noticeable reduction of superficial browning was observed in all cultivars but not always proportionally to treatment time. Textural parameters were affected by plasma treatments only in Red Delicious apples.	**Tappi *et al*. (201**9**)**
Groundnuts	Cold Plasma	13.56 MHz	40 and 60 W at	0 – 30 min	Atmospheric air	1500 and 1950 V	–	–	*A. flavus* and *A. parasitcus*	Results showed complete disintegration of the fungal spore membrane due to electroporation and etching caused by the reactive species of plasma. In 40 W 15 min and 60 W 12 min plasma-treated samples more than 70% and 90% reduction in aflatoxin B1 content was observed	Devi et al. (2017)
Bulk Romain Lettuce	Atmospheric Cold Plasma	0 and 2400 Hz	–	10 min	Atmospheric air	42.6 kV	–	–	*E. coli* O157:H7	More reduction (1.1 log CFU/g lettuce) was observed at the top layer, but shaking the container increased the uniformity of the inhibition. The treatment did not significantly change the surface morphology, color, respiration rate, or weight loss of the samples, nor did these properties differ significantly according to their location in the bulk stack.	Min et al. (2016)
Fresh Cut Melon	Cold Plasma	12.5 kHz	19 V	30 min (15 each side) And 60 min (30 each side)	Air gas	15 kV	–	–	–	Qualitative parameters of fresh-cut melon (soluble solid content, dry matter, color, texture) were only weakly affected. Peroxidase and pectin methylesterase activities were slightly inhibited by the treatment up to respectively about 17 and 7%.	Tappi et al. (2016)
Blue Berries	Atmospheric Cold Plasma	50 Hz	–	2, 5 min	Atmospheric air	60 and 80 kV	–	–	–	Inhibition of pesticides 75.62% − 80.18%	Sarangapani et al. (2016)

#### 3.2.2 In dairy food and dairy food products

Food safety and preservation have become a worldwide issue among food scientists and consumers (Sonawane and Patil, 2020, Ucar et al., 2021). Dairy products are suggested as a nutritious source for individuals of all ages, including children and the elderly, in many nations (Rozenberg et al., 2016, Coutinho et al., 2019a). Milk is considered a complete food due to its nutritional composition and wide range of health advantages (Rathod et al., 2021a). Thermal pasteurization is commonly used to assure milk safety in terms of microbial reduction; nevertheless, this affects the physicochemical and nutritional quality of milk. Furthermore, the pasteurization equipment required a lot of energy to function, which raised the operating costs (Coutinho et al., 2018, Coutinho et al., 2019a). As a result, the application of alternative procedures such as minimum processing, rather than heat treatment, has lately gained popularity (Zhao et al., 2019, Sonawane and Patil, 2020, Rathod et al., 2021a). Therefore, nonthermal technologies such as cold plasma (CP) have emerged as a viable alternative to heat treatment in the dairy industry (Table 3) (Liao et al., 2017a).

**Table 3:** Recent findings summarizing the use of cold plasma in various dairy food and dairy food products.

Food and Food Products	Plasma generating Source	Processing Parameters or Plasma Source							Microorganism	Major Findings and Remarks	Reference
		Frequency (kHz)	Power (W)	Time (min)	Gas	Voltage (kV)	Flow Rate	Pressure			
Milk	DBD	1	–	–	–	32	–	–	–	The degree of protein hydrolysis decreased.	Mohammadpour et al. (2021)
Bovine milk	SD, GD	25	5000	10–30		5–8		1 bar	–	Antigenicity of casein and α-lactalbumin decrease.	Ng et al. (2021)
Milk	DBD	15	80–160	2	–	0.04–0.08	–	–	*S. aureus, E. coli, and L. monocytogenes*	DNA of *S. aureus, E. coli, and L. monocytogenes* destroyed and Metabolic enzyme activity reduced.	Wu et al. (2021)
Dry milk powder	CP	–	480	2	Nitrogen	4.4	8–20 L/min	1 bar	*Cronobacter sakazakii*	*Cronobacter sakazakii* inactivated.	Chen et al. (2019b)
Chocolate milk	CP	50	400	5–15	Nitrogen	–	10–30 mL/min	–	–	SFA content increased and MUFA and PUFA content decreased.	Coutinho et al. (2019a)
Guava flavored whey-beverage	CP	50	400	5–15	Nitrogen	–	10–30 mL/min	–	–	Consistency and viscosity decreased & pH increased.	Silveira et al. (2019)
Bovine milk	JP	13.56 × 10^3^	17	1–12	Helium	–	1.9 L/min	1 bar	*Prototheca zopfii*	*Prototheca zopfii* inactivated.	Tyczkowska-Sieron et al. (2018)
Milk	NPG	2–4	–	05.-2	Argon	9	–		Bacteria	Total bacterial count decreased by 5 log cycles and no changes in the bacterial count for 6 weeks.	Ponraj et al. (2017)
Milk	DBD	15	250	5–10	–	–	–	1 bar	Escherichia coli, Listeria monocytogenes, and Salmonella *Typhimurium*	Reduction in Escherichia coli, Listeria monocytogenes, and Salmonella *Typhimurium* count b 2.4 log cycle.	Kim et al. (2015)
Sliced cheese	DBD	15	250	1–5	–	–	–	1 bar	*Escherichia coli, Salmonella typhimurium, and Listeria monocytogenes*	*Escherichia coli, Salmonella typhimurium, and Listeria monocytogenes* inactivated.	Yong et al. (2015a)

***Abbreviations:*** SFA: Saturated fatty acid; DBD: Dielectric barrier discharge; JP: Jet plasma; NPG: Nanosecond pulse generator; SD: Spark discharge; GD: Glow discharge; CP: Cold plasma.

**Fig. 3:**
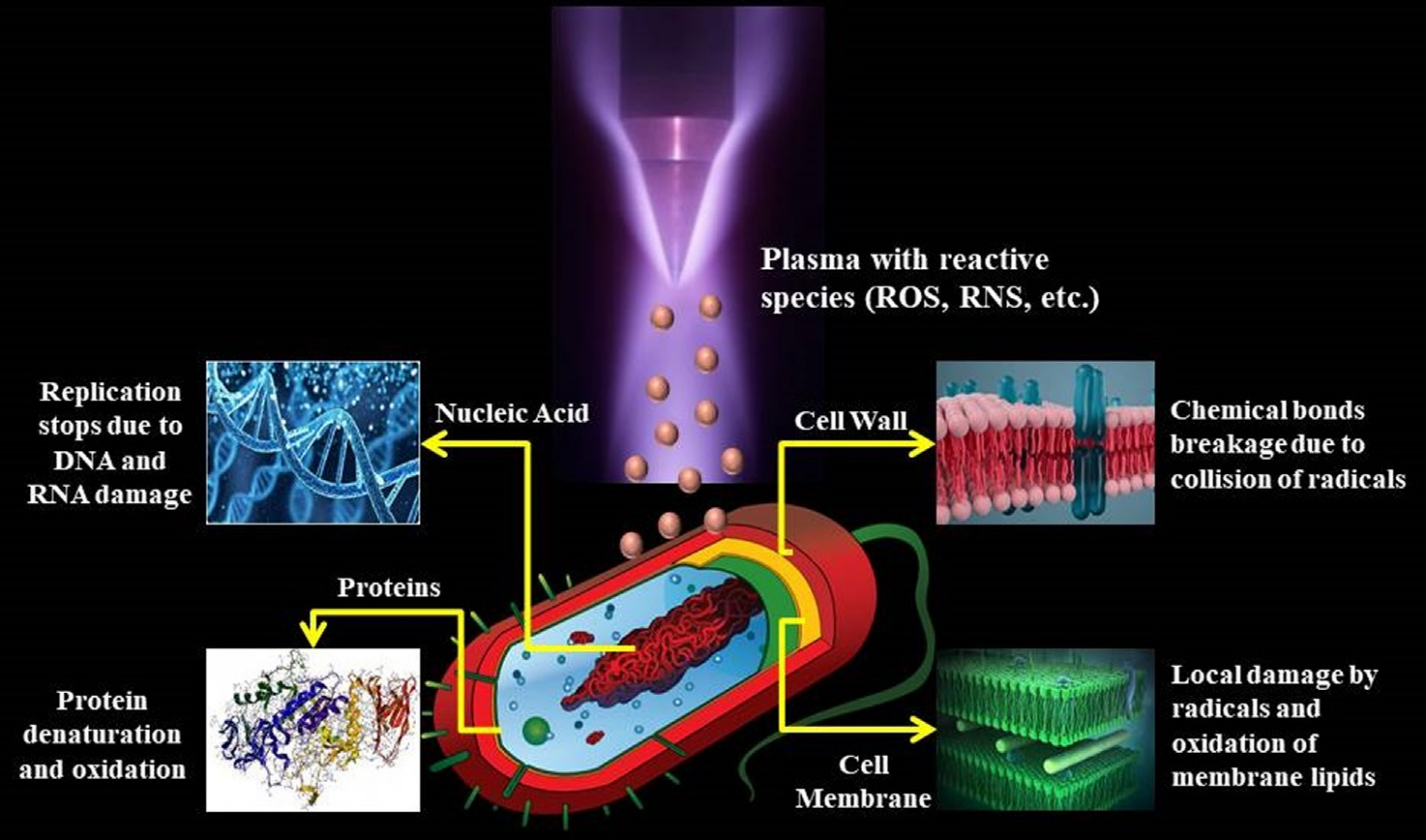
A graphical presentation of antimicrobial action mechanism performs by cold plasma (Deng et al., 2020).

Milk is susceptible to microbial contamination due to its high nutritional value, which is typically caused by bacteria such as *Clostridium, Lactobacillus, Microbacterium, Acinetobacter, Enterobacteriaceae*, and others (Odeyemi et al. 2020). Furthermore, the presence of enzymes in milk, such as protease and lipase, degrades the flavor and quality of milk (Ahmad et al. 2019). Cold plasma is composed of charged particles such as ions and electrons, which influence the microorganism in a variety of ways (Rathod et al., 2021a). A graphical diagram that describes the detailed mechanism of microbial inactivation by cold plasma has been depicted in Fig. 3. The degree of the cold plasma effect is mostly determined by the time of operation. In the instance of *E. coli*, cold plasma is generated high electroporation, which damaged the cell wall and resulted in cell lysis owing to cell constituent leaking (Han et al., 2016b, Timmons et al., 2018). Furthermore, cold plasma efficiently killed spores, which are known to be more resistant to unfavorable environments, by causing cleaves in the genetic components, resulting in the death of the microbial spore (Bermúdez-Aguirre et al., 2013). The flow rate of gases such as argon or helium, as well as the length of treatment, has a significant impact on the inactivation of pathogenic bacteria (Tyczkowska-Sieron et al., 2018, Chen et al., 2019b).

Apart from temperature and light, oxidative deterioration in milk or milk products is caused by the oxidation of unsaturated fatty acids (UFA) by enzymes. The oxidative damage begins with oxygen reacting with free radicals to generate peroxides, which subsequently react with UFA to form ketone aldehydes and alcohols (Rathod et al., 2021a). Cold plasma ROS acts as an oxidant by raising redox potential (Thirumdas et al. 2018). This ROS increases peroxide production in milk; nevertheless, the produced oxidative stress has little effect on antioxidant capacity (Chen et al. 2019b). Lipid oxidation is more prevalent in higher fat milk products. As a result, optimizing fat content and gas composition is required to significantly minimize lipid oxidation (Gavahian et al., 2018, Rathod et al., 2021a).

A change in pH indicates a microbial attack on milk and its products, lowering the milk's quality (Pankaj et al. 2018). Cold plasma treatment decreases the pH of milk due to the generation of hydrogen peroxide and nitric acid by reactive oxygen and nitrogen species (ROS and RNS) (Annapure 2018). In general, factors such as the type of gas, the flow rate, and the operating temperature affect the pH by increasing its acidity (Silveira et al. 2019). When milk was exposed to cold plasma for an extended period, the lightness of the milk decreased as well; however, when milk was exposed to cold plasma for a short period, no change in color was noticed (Chen et al., 2019b, Wu et al., 2020). As a low-cost green technology, cold plasma may be used efficiently to improve the quality of dairy products by eliminating germs. Though the initial cost is higher in cold plasma and is more efficient than heat treatment because it allows for greater control over the process. While the results of cold plasma treatment on dairy products are excellent, the lack of industrial-scale equipment remains a significant issue. Work on the design and implementation of cold plasma devices for the industry can be done in the future.

#### 3.2.3 In meat processing and preservation

Meat and meat products, including beef, pork, fish and poultry, are important human food products because they are considered to be of high quality due to their high protein and nutritional content. Mexico began exporting beef to the Arabian Peninsula in 2017, as Mexican beef is already Halal certified . However, meat and meat products are a nutrient medium for the growth of various pathogenic microorganisms due to their high water content, nutritional composition and pH (Iulietto et al., 2015).

Different types of pathogens are responsible for food damage, such as the degradation of proteins, carbohydrates, fats and other components, which causes food to change color, flavor, smell, and others. As a result of these changes, the appearance of the food undergoes major changes, which means that the food is no longer pleasing to the consumer. On the other hand, the ingestion of meat products contaminated with pathogenic microorganisms causes various types of disease, death, and even large economic losses due to the medical and social costs incurred (Xiang et al., 2017). Meat and meat products are more likely to be contaminated by several bacteria, the most frequent of which are *E. coli O157: H7, Campylobacter* spp.*, Salmonella* spp., *L. monocytogenes*, and others. Various alternatives have been found to keep the meat decontaminated from microorganisms, such as the use of spices, salts, dehydration, freezing, cooling, packaging, and essential oils (Machado-Velasco and Vélez-Ruiz, 2008, Misra and Jo, 2017, Musavu Ndob and Lebert, 2018, Ribeiro et al., 2018). Changes in the structure of the food and loss of firmness are typical after heat treatments are performed on meats, much as oxidation of the lipids leads to rancidity during storage, which is what often happens to chicken flesh (Misra and Jo, 2017). On the other hand, after thermal treatment, unwanted compounds such as polycyclic aromatic hydrocarbons (PAHs), heterocyclic amines, and *N*-nitroso are produced by these processes (Misra and Jo 2017). After plasma treatment, different authors reported positive results. Moutiq et al. (2020) reported a reduction of 2 log CFU/g in the chicken breast of natural microflora (mesophiles, psychrotrophic, and Enterobacteriaceae) at 5 min at 100 kV using ACP and 24 h of storage. The authors ascribed the decrease in natural microflora to reactive oxygen and nitrogen species. While, Ulbin-Figlewicz et al. (2013) treated pork meat with nitrogen, argon, and helium plasma, which reduced the number of psychrotroph bacteria. The total number of microorganisms exposed to helium and argon plasma decreased by around 3 log CFU/cm^2^ and 2 log CFU/cm^2^ for 10 min, respectively. Increased reductions in yeasts and molds were also achieved and were approximately 3 CFU/cm^2^ (helium) and 2.6 CFU/cm^2^ (argon). Table 4 shows the recent findings of some authors who reported the use of cold plasma in various meat products.

**Table 4:** Recent findings summarizing the use of cold plasma in various meat-based food products.

Food and Food Products	Plasma generating Source	Processing Parameters or Plasma Source							Microorganism	Major Findings and Remarks	Reference
		Frequency	Power	Time	Gas	Voltage	Flow Rate	Pressure			
Chicken Breast	Cold Plasma	60 Hz	233 ± 5 W	1, 3 and 5 min	–	100 Kv	–	–	Natural Microflora	2 log CFU/g reduction was achieved within 5 min of treatment and 24 h of storage. After 24, the population of mesophiles, psychrotrophs, and *Enterobacteriaceae* in treated chicken was respectively 1.5, 1.4, and 0.5 log lower than the control.	Moutiq et al. (2020)
Fresh Pork	Low-temperature Plasma	–	450 W	0, 15, 30, 60 s	–	0, 300, 350, 400, 450, 500 W	40 L/min	–	Natural Microflora	The results indicated that the total number of colonies could be reduced by 2 log values under the optimized treatment process (400 W, 30 s)	Zhao et al. (2019)
Boiled Chicken Breast	Atmospheric Dielectric Barrier Discharge Cold Plasma	60 Hz	–	3.5 min	–	38.7 kV (kVRMS)	–	–	*Salmonella*	The concentrations of chicken protein isolate, water, and soybean oil in a chicken breast model food that resulted in the highest Salmonella reduction. ADCP treatment did not affect the color and tenderness of the model food, irrespective of its composition	Roh et al. (2019)
Ready to Eat Ham	Atmospheric Cold Plasma	3500 Hz	300 W	180 s	Sodium Chloride (NaCl)	0–28 kV	–	–	*L. innocua*	Reduction in *L. innocua* of 1.75 and 1.51 log CFU/cm^2^ on 1% and 3% NaCl (4 °C, 180 s). Reduction of *L. innocua* of 1.78 and 1.43 log CFU/cm^2^ (23 °C, 180 s)	Yadav et al. (2019)
Beef Meat	High Voltage in-package Atmospheric Cold Plasma-Dielectric Barrier Discharge	–	–	3–30 min	Atmospheric air	60–80 kV	–	–	–	Plasma induced changes in the functional properties of dairy and beef fat.	Sarangapani et al. (2017)
Egg	Atmospheric Cold Plasma	10e12 kHz	–	–	Helium (He) mixed with chemically active gases: O_2_ and water vapor	25–30 kV	5 L/min	–	*Salmonella enteric*	Reduction of *Salmonella from* 10^8^ to 10^2^ CFU, after 10 min of direct treatment, and 25 min of indirect treatment.	Georgescu et al. (2017)
Fresh mackerel fillet	Atmospheric Plasma	–	–	1, 3 and 5 min	–	70 and 80 kV	–	–	Aerobic psychotropic, *Pseudomonas,* and lactic acid bacteria	Within 24 h of DBD treatment, spoilage bacteria (total aerobic psychotropic, Pseudomonas, and lactic acid bacteria) were significantly reduced.	Albertos et al. (2017)
Pork Meat	Cold Plasma	–	–	5 and 10 min	N_2_, He and Ar	0.8 MPa	–	–	Psychotropic bacteria, Total number of bacteria	Bacteria counts and the total number of microorganisms exposed to He and Ar plasma for 10 min were reduced to about 3 log CFU/cm2 and 2 log CFU/cm^2^, respectively. Increasing reductions of yeasts and molds molds were also obtained and were about 3 cFU/cm^2^ (He) and 2,6 CFU/cm^2^ (argon).	**Ulbin *et al*. (2013)**
Pork Meat	Cold Plasma	–	–	5 s, 2.5 min, 5 min	–	1.2 kW	–	–	Aerobic microbial flor	There was a reduction in the 2 log UFC/g, the samples with no treatment had 9.6 log UFC/g	Fröhling et al. (2012)
Bacon	Cold Plasma	–	–	60 and 90 s	–	75, 100 and 125 W	–	–	*L. monocytogenes* (KCTC 3596), *E. coli* (KCTC 1682), and *S. typhimurium* (KCTC 1925)	After treatment, a microbial reduction of 1.6, 2.0, and 1.5 CFU/G were observed. It is concluded that increasing the treatment time decreases the microbial load more.	Kim et al. (2011)

#### 3.2.4 In seafood and seafood products

At the worldwide level, preserving seafood and ensuring microbiological protection are significant issues. Numerous non-thermal technologies have been developed to accomplish this purpose, most notably cold plasma (Olatunde et al., 2021b). Cold plasma has developed into a highly effective method for satisfying customer demand for stable, nutritious seafood. Seafood is a good source of fat, protein, minerals, and vitamins, and is nutrient-rich. Seafood, on the other hand, has a relatively short shelf life due to its high nutritional content and moisture content (Viji et al., 2017). Rapid biochemical and microbiological changes that occur immediately before death affect the nutritional value and shelf life of seafood (Olatunde and Benjakul, 2018). Seafood is often high in polyunsaturated fatty acids (PUFAs), which increases the likelihood of lipid oxidation (Secci and Parisi, 2016).

Lipids are a critical ingredient in the diet, particularly those high in essential fatty acids. Oxidation of lipids has a detrimental effect on the nutritional and sensory qualities of seafood (Gavahian et al., 2018). The amount of oxygen available, the humidity, the temperature, the amount of light, and the presence of metals all have an effect on the rate of lipid oxidation in seafood (Olatunde et al., 2021b). In seafood, oxidation occurs often as a result of enzymatic or self-oxidation. The ROS and RNS of cold plasma accelerate the oxidation of unsaturated fatty acids (Olatunde and Benjakul, 2020). When cold plasma was applied to Asian sea bass slices, the TBARS (Thiobarbituric acid reactive substances) value increased but the PUFA and monounsaturated fatty acid (MUFA) content decreased (Olatunde et al., 2019). Another aspect affecting lipid oxidation in seafood is the gas composition. Different gases produce a variety of reactive substances, which stimulate lipid oxidation. For example, a combination of argon and oxygen at an 80:20 ratio boosted lipid oxidation more than pure argon air (Shiekh and Benjakul 2020). As reactive chemicals created during cold plasma processing have a detrimental effect on lipid oxidation, it is vital to optimizing the cold plasma process conditions in order to achieve high-quality seafood.

The consumer's acceptance of any food is contingent upon its color, texture, and capability to retain moisture (WHC). Seafood is visually distinctive because of its vibrant colors (Olatunde et al., 2021b). The reactive substances of cold plasma harm the color of meat. The reactive substances oxidized oxymyoglobin (Red) to metmyoglobin (Brown), the primary coloring ingredient in meat ((Sukarminah et al., 2017, Olatunde et al., 2019). Additionally, the gas compositions and voltage of seafood have an effect on its color intensity. The brightness of herring (*Clupea harengus*) diminished significantly with an increase in voltage (Albertos et al., 2017). Generally, cold plasma is utilized for food preservation via dielectric barrier discharge (DBD) and jet plasma (JP) (Olatunde et al., 2021b). Numerous variables, including frequency, voltage, working gas, and time of treatment, all influence cold plasma's antibacterial activity (Olatunde et al., 2019, Olatunde et al., 2020a). When reactive substances are produced during cold plasma treatment, they cause damage to the cell wall of bacteria and leak intracellular components (Olatunde et al., 2021b). This is the most often seen antibacterial activity of cold plasma against a broad spectrum of pathogens. Table 5 summarizes the microorganisms killed by cold plasma in seafood. Additionally, regardless of the kind of reactive substances produced during cold plasma, it is the oxygen-containing compounds that are most responsible for antibacterial action (Olatunde et al., 2021b).

**Table 5:** Recent findings summarizing the use of cold plasma in various seafood and seafood products.

Food and Food Products	Plasma generating Source	Processing Parameters or Plasma Source							Microorganism	Major Findings and Remarks	Reference
		Frequency	Power (W)	Time (min)	Gas	Voltage	Flow Rate	Pressure			
Hairtail (*Trichiurus Lepturus*)	DBD	–	–	0.5–5	–	50 kV	–	1 bar	–	Muscle protein’s texture, water holding capacity, and color were improved.	Koddy et al. (2021)
Blue swimming crab (*Portunus armatus*)	DBD	50	–	0–15	–	80 kVRMS	–	1 bar	*Pseudomonas lundensis, Lysinibacillus macroides, Shewanella baltica, Pseudoalteromonas haloplanktis, Paenisporosarcina quisquiliarum, Pseudoalteromonas aliena,* and *Brochothrix thermosphacta*	Increased the shelf life by decreasing PUFA and inactivating bacteria.	Olatunde et al. (2021a)
Asian sea bass slices (*Lates calcalifer*)		50 Hz		5		80 kVRMS			*Psychrobacter, Pseudomonas, Acinetobacter, Shewanella, Plesiomonas, Enterobacter,* and *Brochothrix*	Bacterial count and lipid oxidation were reduced.	Olatunde et al. (2020a)
Asian sea bass slices (*Lates calcalifer*)	DBD	50 Hz		5		16 kVRMS		1 bar	–	Due to the reduction in trimethylamine content and total volatile nitrogen base content, shelf life was increased to 12–15 days.	Olatunde et al. (2020b)
Threadfin Bream (*Nemipterus bleekeri*)	–	–	30	5–30	Argon	–	–	1 bar	–	Solubility, Ca^2+^-ATPase activity and total SH group content were decreased.	Panpipat & Chaijan (2020)
Pacific white shrimp	DBD	50 Hz	–	10	Argon and oxygen	16 kVRMS	–	1 bar	*Pseudomonas and Enterobacteriaceae*	Shelf life increased to 15 days inactivating the microorganism and Thiobarbituric acid reactive substances and peroxide values were reduced.	Shiekh and Benjakul (2020)
Asian sea bass slices (*Lates calcalifer*	DBD	50 Hz	–	5	Argon and oxygen	16 kVRMS	–	1 bar	–	Shelf life increased to 15 days by reducing Thiobarbituric acid reactive substances and peroxide values.	Singh & Benjakul (2020)
Herring (*Clupea harengus*)	DBD	50 Hz	–	5	–	70–80 kV	–	1 bar	*Pseudomonas, lactic acid bacteria and Enterobacteriaceae*	Microorganisms were inactivated and lipid oxidation was reduced.	Albertos et al. (2019)
Chub mackerel (*Scomber japonicus*)	DBD	50 Hz	–	0.28–1.25	–	10–70 kV	–	1 bar	–	Lipid oxidation was significantly reduced.	Chen et al. (2019c)
Pacific white shrimp (*Litopenaeus vannamei*)	DBD	500 Hz		10		40 kV		1 bar	–	Reduced the bacterial load, and increased the shelf-life to 14 days.	De Souza Silva et al. (2019)
Squid (*Argentinus ilex*)	DBD	–	–	0.2–5	–	60 kV	–	1 bar	–	Texture, color and water holding capacity of the treated squid gel increased.	Nyaisaba et al. (2019)
Hairtail (*Trichiurus japonicus*)	DBD	–	–	2.5–15	–	30–50 kV	–	1 bar	–	Decreased the endogenous enzyme activity.	Hatab (2018)
Mackerel (*Scomber scombrus*)	DBD	–	–	1–5	–	70–80 kV	–	1 bar		Microbial load and lipid oxidation were decreased.	Albertos, et al. (2017)

### 3.3 Relation of food functionality and cold plasma

Functional compounds are a class of substances that include essential nutrients and are produced from plants or plant-based foods, such as proteins, starches, phenolic compounds, and carotenoids (Pereira et al., 2020). However, because these functional molecules are heat-sensitive, conventional food processing destroys them (Khan et al., 2018). As a result, nonthermal processing methods are gaining popularity since they produce higher-quality and nutritionally rich foods (Iqbal et al., 2019). Cold plasma (CP) is a nonthermal processing technology that has a variety of uses in food processing, including the alteration of food product functionality (Table 6) (Rathod et al., 2021b, Sruthi et al., 2021).

**Table 6:** Recent finding summarizing the effect of cold plasma on the functionality of food.

Food and Food Products	Plasma generating Source	Processing Parameters							Effect on Functionality	References
		Amount of Sample	Frequency (Hz)	Time (min)	Gas	Voltage (kV)	Flow Rate	Pressure		
Tender coconut water	DBD	–	50	1–3	–	18–28	–	1 bar	Total phenolic content and ascorbic acid content decreased.	Chutia & Mahanta (2021)
Sour cherry juice	JP	5–15 ml	10–20	1–9	1% oxygen gas in argon gas	10–20	3 L min^−1^	1 bar	Total phenolic content increased.	Hosseini et al. (2021)
Cashew apple juice	DBD	20 ml	200 & 700	15	–	20	–	1 bar	Vitamin C content increased. No changes were noticed in phenolic contents.	Leite et al. (2021)
Kiwi turbid juice	DBD	10–20 ml	60	1–5	–	10–40	–	1 bar	Total phenolic content decreased. Flavour and texture improved.	Liu et al. (2021)
Powdered Spirulina algae	DBD	5 mg	10	0–5 min	–	–	–	1 bar	The color value decreased.	Beyrer et al. (2020)
Wheat flour	DBD	1 mg	50	5–30	–	80	–	1 bar	Hydration property increased. WSI decreased.	Chaple et al. (2020)
Dried peppermint	Radio-frequency LPCP	–	13.56 × 10^6^	20	Pure oxygen	–	–	40 mTorr	Total phenolic content decreased.	Kashfi, et al. (2020)
Green coconut water	DBD	20 ml	200–730	15	–	15–20	–	1 bar	No changes were found in phenolic content.	Porto et al. (2020)
Strawberry	DBD	15–18 g	50	10–30	–	60	–	1 bar	Total soluble solid increased.	Rana et al. (2020)
Blueberry juice	JP	–	1000	2–6	Argon and oxygen	11	–	1 bar	Anthocyanin and antioxidant activity decreased.	Hou et al. (2019)
Fresh-cut pitaya	DBD	–	–	5	–	60	–	–	Total phenolic content and antioxidant activity increased.	Li et al. (2019)
Siriguela juice	GDP	80 ml	50 × 10^3^	5–15	Nitrogen	–	10–30 ml min^−1^	–	Carotenoid content increased.	Paixão et al. (2019)
Tomato juice	GAD	5 ml	50	0.5–5	Nitrogen	3.8	7.33 L min^−1^	1 bar	Vitamin C content decreased.	Starek et al. (2019)
Wheat	GAD	–	200	3–15	–	4–6	–	1 bar	Water permeability increased	Roy et al. (2018)
Pumpkin puree	CD	25 g	–	5–20	–	17	–	–	Carotenoid content decreased.	Santos et al. (2018)
Wheat Germ	DBD	–	50	5–35	–	24	–	1 bar	No changes were reported in phenolic content.	Tolouie et al. (2018)

***Abbreviations:*** DBD: Dielectric barrier discharge; JP: Jet plasma; LPCP: Low-pressure cold plasma; GDP: Glow discharge plasma; GAD: Gliding arc discharge; CD: Corona discharge.

**Fig. 4:**
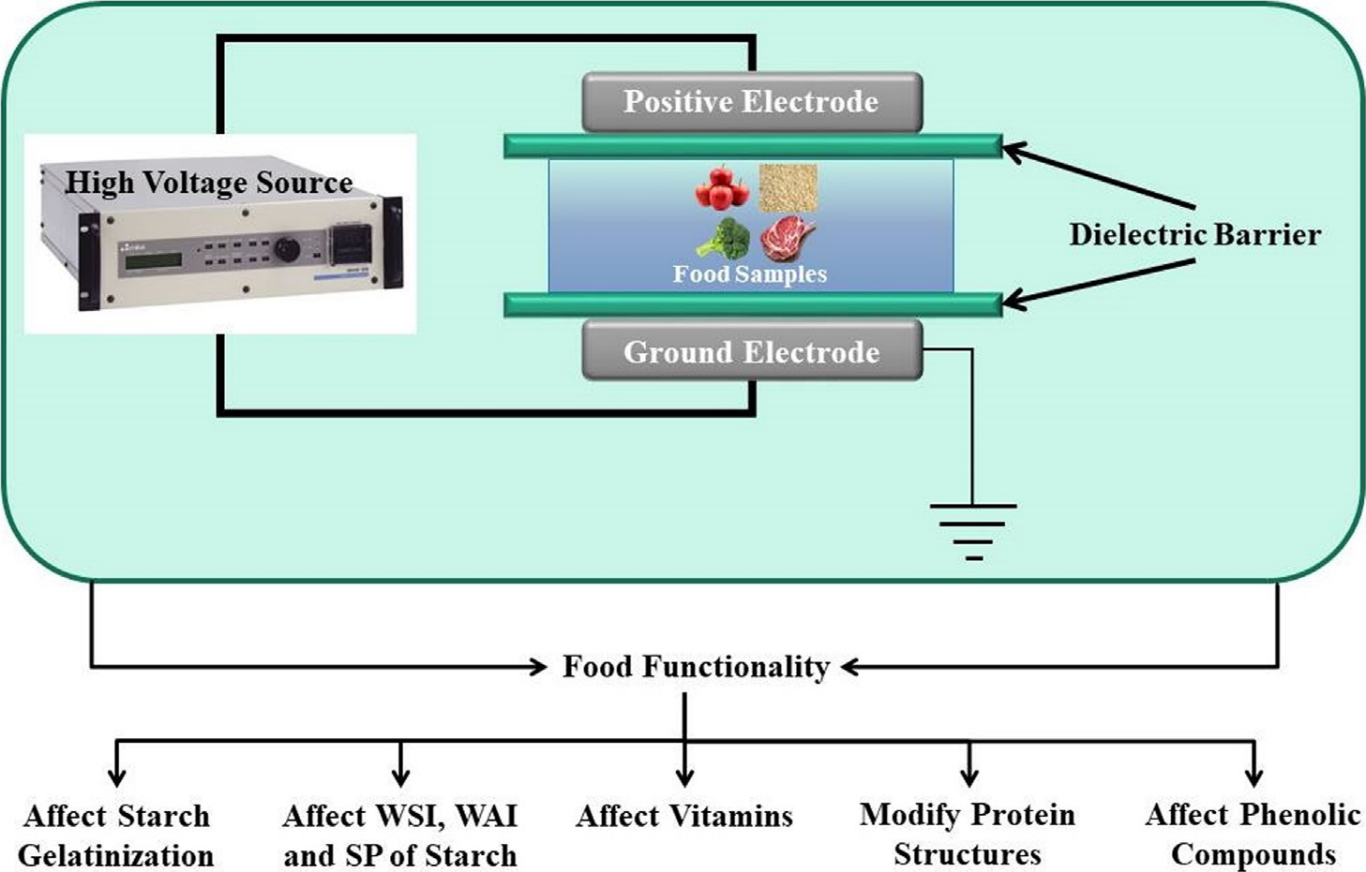
Effect of cold plasma on the functionality of various foods.

Among the different components of foods such as cereals, starch is the most significant and prevalent. Starches are widely utilized in the production of a variety of food products (Bian et al., 2021, Sruthi et al., 2021). Thermal processing, on the other hand, affects its functioning by starch gelatinization, as evaluated by several qualities such as swelling power (SP), water absorption index (WAI), and water solubility index (WSI), among others (Chaple et al., 2020, Gandhi et al., 2021). Plasma processing has been shown to modify the native ingredients of foods in beneficial ways. Plasma-treated rice flour had a greater WAI with an increase in plasma power and application time (Thirumdas et al., 2016). This occurred as a result of the depolymerization of starch produced by the interacting plasma species. The impact energy and mean velocity of nitrogen molecules rise, resulting in an increase in starch damage (Thirumdas et al., 2017). The use of a low concentration of cold plasma altered the functioning of wheat flour and resulted in a strong dough (Bahrami et al., 2016). Plasma-treated food has a rougher surface morphology, which increases the effective contact area and hence the wettability (Roy et al., 2018, Chaple et al., 2020), resulting in a reduction in cooking time (Thirumdas et al., 2016). Plasma generates fissures in the outer layer, which aided in the leaching of amylose throughout the rice cooking process, altering the rheological and solubility of cooked rice (Sruthi et al., 2021). Additionally, the swelling of amylopectin influences the other non-starch components, which in turn impacts the swelling of granules due to amylose–lipid complexes (Khatun et al., 2019, Sruthi et al., 2021). The findings show that cold plasma processing may significantly alter the characteristics of foods and can be customized to regulate their functionality (Fig. 4). However, there are just a few publications on the molecular-level interaction between cold plasma with flour starch.

Protein is the second most essential component of the food system, after starch, and its functional qualities are defined by the intricate interactions between the structures (Mollakhalili-Meybodi et al., 2021). While the primary structure has the greatest influence on functionality, secondary and tertiary structures are equally critical (Akharume et al., 2021). Cleavages in amino acid chains, notably in NH or NH2 or peptide bonds, resulting in increased carbonyl components when cold plasma is applied to whey protein isolates (Segat et al., 2015, Liu et al., 2020). Additionally, after cold plasma treatment at 60 kV for varied application periods, a drop in sulfhydryl groups and a rise in carbonyl content was seen in crude protease (Nyaisaba et al., 2019). The reactive species can alter the activity of proteins directly or indirectly by activating the nearby components (Mollakhalili-Meybodi et al., 2021). Thus, the matrix to which proteins are exposed is critical because it has the potential to influence their functioning (Misra et al., 2016, Mollakhalili-Meybodi et al., 2021).

Phenolics, which are found in fruits and vegetables, are helpful to human health as the principal antioxidant compound (Issaoui et al., 2020). They possess a variety of beneficial qualities, including antioxidant, antimicrobial, and anti-inflammatory capabilities, which aid in the preservation of food quality (Yaqoob et al., 2020, Sruthi et al., 2021). However, it was noted that when cold plasma was applied to several foods, such as apple juice, white grape juice, tomato juice, acerola juice, and chocolate milk, the concentration of phenolic compounds dropped (Pankaj et al., 2017, Liao et al., 2018, Chen et al., 2019a, Coutinho et al., 2019a, Coutinho et al., 2019b, Fernandes et al., 2019, Ali et al., 2021). Plasma parameters such as power, exposure time, and flow rate all have a direct effect on the phenolic content of food (Sruthi et al., 2021). The production of ozone following plasma discharge reduced the concentration of phenolic compounds by acting on the aromatic rings of phenolic compounds. As a result, phenolic compounds were sensitive to ozone assault, with substantial changes occurring only after 60 s of plasma exposure (Almeida et al., 2015, Sruthi et al., 2021). Because a thorough and in-depth understanding of the impacts of plasma-responsive species on food activity is still in its infancy, the discipline requires more investigation. The discrepancy between the two studies demonstrates the importance of conducting more systematic research to better understand the interaction between active food components and plasma-reacting species.

## 4 Important methods and factors influencing cold plasma efficiency

**Fig. 5:**
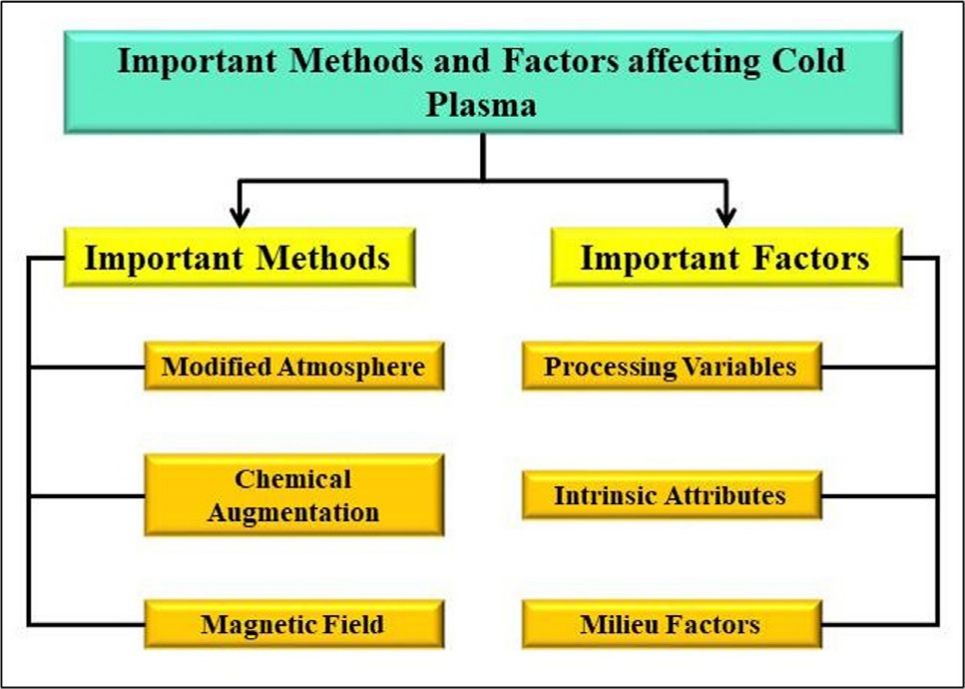
Methods and factors that impact the efficiency of cold plasma.

Plasma sometimes referred to as the fourth state of matter, is an ionized plasma composed of charged particles, free radicals, and a small amount of radiation. It is formed with an electrical discharge, which produces a partially or completely ionized plasma composed mostly of photons, ions, free electrons, and atoms (Coutinho et al., 2018). Plasma is generally neutral in nature, which means that the number of positive charges equals the number of negative charges (Ekezie et al., 2017). The efficiency of plasma treatment can be increased by modifying several parameters such as the feeding gas, the plasma source, the input voltage, and frequency, or by combining plasma with other technologies (Fig. 5), all of which are discussed in brief in the following section.

### 4.1 Important methods

When cold plasma treatment is used, the plasma characteristics are highly dependent on the parameters of the gas medium. Thus, the type of gas utilized in cold plasma is crucial, as various gases have varying effects, such as varying rates of microorganism inactivation (Feizollahi et al., 2021). Additionally, food products are packed with a variety of gas compositions to increase their shelf life, a process called modified atmospheric packaging (MAP) (Ekezie et al., 2017). For example, *Salmonella enterica* was reduced by 2.9 logs in the air and by 4.7 logs in a modified atmosphere of 65% O_2_ + 30% N_2_ + 5% CO_2_ when applied directly to an orange juice sample for 120 s and then stored at 4 °C for 24 h (Xu et al., 2017). *Escherichia coli, Staphylococcus aureus,* and *Listeria monocytogenes* were all demonstrated to be affected by MAP gas combinations during inactivation (Han et al., 2016a). After cold plasma treatment, bacteria like *Campylobacter jejuni* were more effectively inactivated by air than by nitrogen. The presence of oxygen and nitrogen, followed by nitrogen alone, is strongly suggested during cold plasma treatment to accomplish microbial inactivation (Kim, et al. 2014). During cold plasma treatment of gas mixtures, oxygen, ozone, and other antimicrobially active species such as singlet oxygen, superoxide ions, and hydroxyl radicals can be generated (Ekezie et al., 2017, Feizollahi et al., 2021). Hence, ROS (such as ozone and peroxides) are formed when there is oxygen in the environment (Shi et al., 2017). This leads to microbial inactivation. Protein breakdown, fragmentation, and release of DNA, and cell membrane oxidative damage can be caused by ACP reactive species. Apoptosis and distortion of mycelial tips in fungi are both caused by intracellular oxidative stress (Liao et al., 2017a, Misra et al., 2019).

The addition of plasma-activated species to a product, such as a hydrogen to water (plasma-activated water; PAW), falls under the category of chemical augmentation (Ekezie et al., 2017, Herianto et al., 2021). This PAW is capable of killing a variety of pathogenic microbes, including *E. coli* (Royintarat et al., 2019, Xiang et al., 2019, Zhou et al., 2018), *S. aureus* (Royintarat et al., 2019, Vlad et al., 2019, Xiang et al., 2019), and *Pseudomonas deceptionensis* (Xiang et al., 2018). After 15 min in 500 ml of PAW, mushrooms demonstrated 1.5 and 0.5 log decrease in bacterium and fungal populations, respectively, with delayed softening (Xu et al., 2016). Many researchers have claimed that PAW’s antibacterial activity contributed to the synergistic effects through its physicochemical characteristics, including ROS, RNS, pH, and UV radiation. However, the most important components and their specific mechanism are still under contention (Thirumdas et al., 2018, Herianto et al., 2021). Sodium dodecyl sulfate (SDS) and lactic acid applied with cold plasma together with PAW reduced the activity of *E. coli* and *L. monocytogenes* by 3.77 and 4.78 log CFU/cm^2^, respectively (Trevisani et al., 2017). These studies show that substances such as food-based sanitizers or natural antimicrobials can improve cold plasma efficiency.

Another approach is the use of a magnetic field with cold plasma to increase the density of the plasma (Ekezie et al., 2017). By applying 0.587 T magnetic field to a plasma jet, the concentration and electron density of the cold plasma grew by 2.4 and 1.5 times, respectively (Liu et al., 2016). Hydroxyl radicals were also shown to rise, and the plasma's ability to kill *E. coli* was boosted by 1.23 times (Liu et al., 2016). Biomedical applications and culinary applications may leverage this method in the future to boost radical generation at the plasma/substrate interface.

### 4.2 Important factors

Cold plasma processing variables such as voltage, frequency, current, and electric field strength all have a favorable impact on the treatment (Ekezie et al., 2017, Feizollahi et al., 2021). The rate of microbial inactivation rose as the voltage, power, and frequency of cold plasma increased (Albertos et al., 2017, Liao et al., 2018, Wang et al., 2018). Ozon, a potent oxidizing agent and primary ROS produced by cold plasma, is employed in water disinfection. When the applied voltage was raised from 55 to 80 kV, the ozone concentration increased from 200 to 950 ppm, which eventually harmed the microorganisms (Wang et al., 2018). Furthermore, the mode of exposure has an effect on cold plasma efficiency and is preferable to indirect or remote exposure when trying to improve process efficiency due to the lower heat transfer rate to the matrix when considering charged particles’ self-quenching nature and recombination abilities before they reach the sample (Patil et al., 2014, Ekezie et al., 2017). Another process variable that impacts the effectiveness of cold plasma is the treatment time. The prolonged exposure to cold plasma produces a higher amount of ROS, which enhances the death of microorganisms at higher inactivation rates (Albertos et al., 2017, Liao et al., 2018, Xu et al., 2017, Zhang et al., 2017b). The duration of cold plasma treatment was extended from 5 to 45 s, which resulted in an increase in the mortality rate of *S. aureus* from 0.09 to 4.95 log CFU/mL (Liao et al., 2017b). However, a longer treatment period may have an effect on the product's quality. As a result, processing time should be reduced.

The type of microorganism and its properties also affect the efficiency of the cold plasma. Microbial type, strain, and mode of existence all have a role in the sensitivity of yeasts and molds to cold plasma treatment compared to mesophilic bacteria (Lunov et al., 2016, Los et al., 2018). DBD ACP treatment of tomatoes decreased mesophilic aerobic bacteria by 1.3 log CFU/tomato, whereas yeast and molds by 1.5 log CFU/tomato (Min et al., 2018). Additionally, when bacteria are in the exponential phase, they are less susceptible to cold plasma than when they are in the stationary phase, and gram-negative bacteria are more sensitive due to the thickness of their lipopolysaccharide membranes and peptidoglycans (Lunov et al., 2016, Ekezie et al., 2017). By and large, bacterial spores are more resistant to plasma than vegetative cells (Ekezie et al., 2017, Feizollahi et al., 2021). Furthermore, because of the intricate stiffness conferred by the chitin-based cell walls of fungus, they are more resistant to plasma treatment than bacteria (Liang et al., 2012, Ekezie et al., 2017). It was found that the order of susceptibility of various microorganisms to ACP is yeast-mold-virus > bacteria > spores (Feizollahi et al., 2021).

Numerous environmental variables, such as relative humidity (RH), temperature, and pH, all influence the efficiency of cold plasma treatment. The rate of microbial activation increased as RH increased, owing to the increased concentration of OH, the most effective ROS at high RH (Feizollahi et al., 2021). When the discharge has a high level of humidity, electron energy can be lost in electron-molecule collisions, weakening the plasma (Butscher et al., 2016). Another factor, pH, inhibited microbial development more rapidly in acidic conditions, as demonstrated by the decrease of Bacillus cereus by 4.7 logs at pH 5 and 2.1 logs at pH 7 (Ekezie et al., 2017). Due to the low temperature at which cold plasma is generated, the thermal process has a negligible effect on microbial inactivation.

## 5 Safety aspects, regulation and challenges in cold plasma

Food safety is critical for every technique of food processing. Cold plasma has been examined for its toxicological effects on food and packaging materials. The modified starch contains no new components as a result of the cold plasma modification, as confirmed by the FTIR investigation (Sifuentes-Nieves et al. 2021). This demonstrates the safety of cold plasma in the modification of the starch process. Although the cold plasma generated several ROS, ozone is the primary ROS with antibacterial action. Therefore, the United States-Food and drug administration (US-FDA) has established specific rules and regulations governing the quantity of ozone in plasma; however, no such standards exist for the other ROS present in cold plasma (Cullen et al., 2018). Additionally, the toxicological impact of cold plasma on the edible film was investigated in male and female Sprague–Dawley rats, and it was determined to be negligible (Han et al. 2016c). In general, cold plasma is harmless under typical working circumstances. For industrial uses, however, each operating system and generated food must be thoroughly inspected for the presence of any dangerous chemicals.

At the worldwide level, regulatory implications of emerging technology vary. In the United States, the Food and Drug Administration (FDA), the Environmental Protection Agency (EPA), and the United States Department of Agriculture (USDA) have all authorized the use of cold plasma in food and food packaging. In agriculture or food processing, it is sufficient to have minimal evidence (or scientific data) that the application of atmospheric cold plasma provides continuous treatment for the most extreme process conditions without endangering humans, the environment, the economy, or society, as defined by the Federal Food, Drug, and Cosmetic Act 408. Additionally, the USDA-FSIS (USDA-Food Safety Inspection Service) must authorize any future use of atmospheric cold plasma in meat, poultry, or eggs. Novel foods should be permitted to be used if they do not endanger public health, have no adverse effect on nutrition and do not mislead consumers. The European Food Safety Authority's (EFSA) expert scientific panel gathers the necessary data and then provides an expert report on the positive and negative impacts of novel foods. Only novel foods that have been authorized by the EFSA should be permitted to be sold on the market.

Various problems that may be converted into possibilities in the use of cold plasma can only be overcome by learning about reactive plasma species and purification procedures. Plasma impacts negatively on the color and nutritious composition of beverages. Because color is a fundamental factor for food and beverage consumption, researchers have a significant problem in maintaining color during cold plasma treatment. Furthermore, for industrial applications, the cold plasma source must be designed, developed, and installed in such a way that it does not interfere with the current process line. A reduced sample quantity or volume is required for laboratory work. In practice, however, the processing needs are significant. As a result, for smooth and continuous functioning, adequate volumetric scale-up is essential. Maintaining plasma uniformity is also necessary during scale-up, which necessitates the design of a suitable plasma source that can match the plant's required capacity.

## 6 Existing patent work on plasma technology

More than 1 000 000 patent issued worldwide, of which some 750 000 have not expired, was found in a patent review using the terms “plasma,” “cold plasma,” “non-thermal plasma” and “gas discharge.” If the patent is randomly divided into 50:50 between fusion-related plasmas and fusion-unrelated to plasmas, a wide body of accumulated intellectual property (IP) is present around non-thermal technology plasmas that can be put on the market (Weltmann et al., 2019). This is also valid since it is accepted that a large number of patents have already resulted in commercial applications in the field of non-thermal plasma processing for semiconductors and microprocessors. Certain products and processes that have benefited, perhaps to a lesser extent, from the promotion of existing plasma-related IPs but still have considerable untapped market opportunities in this respect included intense UV excimer lamps, material processing, surface alteration, nanoparticle synthesis, ozone development, and environmental recovery (Weltmann et al., 2019).

Going back to the history of the 1960s, for the first time, plasma sterilization property was introduced and a patent was filed in 1968 (Menashi, 1968). Surfaces such as glass, plastics, and ceramics were treated with plasma to make them sterile free from microorganisms (Menashi, 1968). The method involves the exposure of the surface to plasma for sufficient time to destroy microorganisms without affecting the physical properties of the surface. Sterilization is achieved by exposing the surface to be sterilized to plasma for a very short period of time, usually not longer than one-tenth of a second. To prove this, glass microscope slides with different concentrations of bacteria were introduced on the slide and concentrations up to 4 × 10^6^ spores per square inch were found to have been completely destroyed in <1/10 of a second. Menashi (1968) pointed out that the actual voltage required to form the plasma will depend on several factors, including the sharpness of the discharge point, the frequency of the current supplied, the gas used to form the plasma, and the volume defining the plasma. This was just the beginning of different cold plasma-related work in different industries. Many people like L. E. Ashman, and W. P Menashi, (Ashman and Menashi, 1972), R. M. G. Boucher (Boucher, 1980), and R. M. Bithell (Bithell, 1982) subsequently filed a number of patents claiming that electrical discharges would lead in particular to the complete sterilization of gasses. The killing of 106 spores in the internal surface of the vials by pulsed RF field plasma occurred in less than a second with argon plasma. Boucher (1980) clarified how UV radiation plays an important role in microbial plasma inactivation and confirmed that only one micrometer of UV photon can penetrate to depth, while the plasma can penetrate 10 µm to allow the removal of sporulated bacteria. Jacobs and Lin (1987) subsequently used H_2_O_2_ as a sterilizer and used plasma to remove chemicals’ residues from sterilized products. Nelson and Berger (1989) described the harmful behavior of plasma combined with oxygen (O_2_) on the biological matter. The researchers found effective biocidal action on *Clostridium sporogenes* and *B. subtilis* by oxygen plasma as both were known to be the most resistant bacteria. Plasmas produced at 200 W were enough to reduce the *B. subtilis* population by more than 3.5 log10 in 5 min (Hury et al., 1998). The use of plasma to sterilize was promoted ever since then (Laroussi, 1999).

Cold plasma is effectively used for sterilization and packaging alteration of polymers, but it is commonly used in food processing. Most scientists have successfully used plasma for microbial inactivation in foods (solids and liquids), but their effects on nutritional properties and food toxicity have not been explained. Bompeix and Sardo (1999) patented the process for the treatment of cold plasma fruits and vegetables applied to bulbs, flowers, mushrooms, potatoes, onions, seeds, tubers, and, in particular, to the coating of apples and pears. They developed a process in which fruit and/or vegetables were first treated with physical treatment, optionally followed by cold plasma, with the aim of improving the effectiveness of the coating materials on the fruit and/or vegetable surface. Plasma was generated under room conditions where the temperature did not rise above 40 °C. Plasma use may also result in minimum levels of fungicides, pesticides, bactericides, or other preservatives. Later, Herrmann and Selwyn (2001) developed a plasma atmospheric-pressure decontamination/sterilization chamber. They were used to decontaminate or sterilize medical and food-related equipment and substances where materials were to be decontaminated or sterilized. In the chamber, the materials were stored in the reactive gases of atomic and metastable oxygen species, formed in the He/O_2_ mixture by atmospheric pressure plasma discharges, which led to a chemical process between the reactive species and organic substance. This process normally destroyed and/or neutralized the contaminants without damaging most of the equipment and materials and circulated the plasma gases. In another sterilization patent, Paskalov (2013) described the plasma sterilization systems and methods that included the sterilization of a substance to be sterilized into a rotating chamber and the plasma irradiation of a substance produced from a radiofrequency (RF) generator. Gas with at least 85 % N_2_ included a type of NOx that is effective in killing microbial organisms. This method was well suited to the sterilization of food powders and other food-based materials, which are not possible in other methods. Paskalov (2014), in accordance with the previous patent, again developed plasma sterilization systems and methods but this method promoted the sterilization of substances (inside the chamber) and RF plasma by generating the magnetic field that gave the substance force to the direction of the chamber in opposition to the rotating direction. The cell had a gas-permeable wall in other respects and the substance was subject to the waves of the acoustic shock produced through a modulating RF generator. A patent on the use of cold plasma to kill or reduce pathogens or denature proteins in food systems has recently been published by M. C. Jacofsky and G. A. Watson (Jacofsky and Watson, 2016). Plasma was directed from a cold plasma device to a food or a food surface over an effective area for an effective amount of time and a cold plasma device can be a DBD (boron-doped diamond) electrode device or an army of DBD electrode devices.

Thus, several patents on the use of cold plasma in food processing industries have been published. Previously, most patents were based on the set-up, system, and methodology of cold plasma devices, but recent research into the application of plasma to food processing and preservation has increased, reflecting an increased number of patents from the past 15 years. It can therefore be highly recommended that the use of plasma in food must be recognized as GRAS following comprehensive work and studies in this area (in vitro and in vivo). Future research should be undertaken to make cost-effective use of plasma on food surfaces to alter its physical and chemical properties.

## 7 Perspectives and opportunities of plasma in the food processing industry

Cold plasma technology has a wide range of opportunities in the food processing and preservation sector. However, its successful implementation would face a number of challenges. For its applications, the species of reactive plasma, the mechanism of inactivation, equipment limitations, and other adverse effects must be understood. Cold plasma is currently being extensively explored for its potential for higher-efficiency pasteurization of food products. It meets all consumer requirements because it maintains the quality of food and satisfies consumer demand for safe, healthy, appetizing, inexpensive, low-processing, and longer shelf-life food (Mir et al., 2016).

Conventional thermal treatments such as pasteurization and sterilization inactivate microorganisms and enzymes to produce safe and high-quality food products with improved shelf-life, but the intensity of the pasteurization process (time and temperature) must be regulated (Aghajanzadeh and Ziaiifar, 2018). In order to prevent contamination by pathogenic microorganisms, avoid the generation of toxic substances and changes in taste in milk, the raw material should be subjected to thermal processes prior to its commercialization (Coutinho et al., 2018). Korachi et al. (2015) analyzed changes in milk bronchus following a cold plasma treatment for *E. coli* decontamination using 9 Kv of power at different time intervals of 0, 3, 6, 9, 12, 15, and 20 min. After three minutes of cold plasma treatment, a 54% reduction in microbial load was observed. Further research by Kim et al. (2015), who used 250 W, 15 kHz plasma treatment over a period of 5–10 min for decontamination of inoculated milk with *S. typhimurium, E. coli,* and *L. monocytogenes* with an initial concentration of 6.28, 6.43 and 6.21 CFU/mL, which decreased to 2.43, 2.40 and 2.46 CFU/mL after plasma treatment, respectively. In addition to milk, cold plasma has also been used to decontaminate different juices such as orange, tomato, and apple inoculated with *E. coli* (Dasan and Boyaci, 2017). Dasan and Boyaci (2017) used cold plasma treatment at 650 W for 30, 60, 90, and 120 s and, following treatment, a reduction of *E. coli* to 1.59 ± 0.17, 1.43 ± 0.22 were observed from the initial concentrations of 4.02 ± 0.03 and 3.34 ± 0.09 CFU/mL without affecting the color or flavor of the products. Hou et al. (2019) used 0, 0.5%, and 1% of oxygen concentrations for time 2, 4, and 6 min and reported a 7.2 log reduction in *Bacillus* cells in blueberry juice as treatment time and oxygen concentration increased. However, the increase in treatment time resulted in a significant (p < 0.05) decrease in anthocyanin, vitamin C, and antioxidant activity. They also reported a decrease in vitamin C content and% OH free radical from higher oxygen concentration, while TPC, antioxidant activity in DPPH and ABTS assays increased with oxygen concentration. The authors were concluded that cold plasma processing had a more positive effect on the quality of blueberry juice compared to thermal processing.

More food quality and safety-based research are therefore needed to assess the effect of cold plasma in food preservation and processing, in particular on microbial inactivation and its nutritional impact. However, researchers are looking at cold plasma in food processing as a safe technology, but more experiments need to be conducted comprehensively to understand the effect of plasma on the generation of toxic substances or undesired chemical changes. It is considered as a chemical-free process till the date and is also ideal for further research on organic food processing. Researchers must focus on scale-up studies of cold plasma systems and standardize factors such as power density, carrier gas, voltage, frequency, gas flow, etc. for commercial application. In the coming years, they should also explore cold plasma as part of “hurdle technology”.

## 8 Concluding Remarks

Cold plasma technology is a relatively new technique in food processing and preservation, but is a promising alternative to traditional methods, considering higher processing efficiency and minimal changes in food quality. These benefits may pave the way for food products, which are highly desirable for consumers but are still in the early stages of their commercialization. Several advantages, such as decontamination of food products, minimal changes in food quality, no wastewater, environmentally friendly and reliable plasma technology, make it attractive to the food industry. However, the literature on the optimization of industrial processes is still limited and unclear. We therefore strongly recommend that extensive research should be carried out in the future to work at an industrial level and on a large scale. Another issue is the need for significant multi-directional research, such as starting with plasma reactors, to ensure efficient, large-scale, and uniform processing and to continue with process optimization in terms of materials, precursors, gas flows, and process conditions. Furthermore, cold plasma still requires further study in order to establish the process for a number of food products, to understand the mechanism of action and other unknown factors which are essential in optimizing the process for separate applications.

## Declaration of Competing Interest

The authors declare that they have no known competing financial interests or personal relationships that could have appeared to influence the work reported in this paper.

## References

[b0005] Afshari R., Hosseini H. (2014). Non-thermal plasma as a new food preservation method, Its present and future prospect. J. Paramed. Sci. Wint..

[b0010] Aghajanzadeh S., Ziaiifar A.M. (2018). A review of pectin methylesterase inactivation in citrus juice during pasteurization. Trends Food Sci. Technol..

[b0015] Ahmad T., Butt M.Z., Aadil R.M., Inam-ur-Raheem M., Bekhit A.E.D., Guimarães J.T., Cruz A.G. (2019). Impact of nonthermal processing on different milk enzymes. Int. J. Dairy Technol..

[b0020] Akharume F.U., Aluko R.E., Adedeji A.A. (2021). Modification of plant proteins for improved functionality: A review. Compr. Rev. Food Sci. Food Saf..

[b0025] Alam A., Wan C., McNally T. (2017). Surface amination of carbon nanoparticles for modification of epoxy resins: plasma-treatment vs. wet-chemistry approach. Eur. Polym. J..

[b0030] Albertos I., Martin-Diana A.B., Cullen P.J., Tiwari B.K., Ojha K.S., Bourke P., Rico D. (2019). Shelf-life extension of herring (*Clupea harengus*) using in-package atmospheric plasma technology. Innovative Food Sci. Emerg. Technol..

[b0035] Albertos I., Martín-Diana A., Cullen P.J., Tiwari B.K., Ojha S.K., Bourke P., Rico D. (2017). Effects of dielectric barrier discharge (DBD) generated plasma on microbial reduction and quality parameters of fresh mackerel (*Scomber scombrus*) fillets. Innovative Food Sci. Emerg. Technol..

[b0040] Al-Hilphy A.R.S., Verma D.K., Niamah A.K., Billoria S., Srivastav P.P., Meghwal M., Goyal M.R. (2016).

[b0045] Ali M., Cheng J.H., Sun D.W. (2021). Effects of dielectric barrier discharge cold plasma treatments on degradation of anilazine fungicide and quality of tomato (*Lycopersicon esculentum* Mill) juice. Int. J. Food Sci. Technol..

[b0050] Almeida F.D.L., Cavalcante R.S., Cullen P.J., Frias J.M., Bourke P., Fernandes F.A., Rodrigues S. (2015). Effects of atmospheric cold plasma and ozone on prebiotic orange juice. Innovative Food Sci. Emerg. Technol..

[b0055] Annapure U.S. (2018). Application of cold plasma in food processing. Technol. Food Process..

[b0060] Ashman, L.E., Menashi, W.P., 1972. Treatment of surface with low-pressure plasmas.. U.S. Patent No. 3,701,628. U.S. Patent and Trademark Office, Washington, DC.

[b0065] Athukorala S.N.P., Fernando W.G.D., Rashid K.Y. (2009). Identification of antifungal antibiotics of *Bacillus* species isolated from different microhabitats using polymerase chain reaction and MALDI-TOF mass spectrometry. Can. J. Microbiol..

[b0070] Ayhan Z. (2017). Minimally Processed Refrigerated Fruits and Vegetables.

[b0075] Bahrami N., Bayliss D., Chope G., Penson S., Perehinec T., Fisk I.D. (2016). Cold plasma: A new technology to modify wheat flour functionality. Food Chem..

[b0080] Bauer A., Ni Y., Bauer S., Paulsen P., Modic M., Walsh J.L., Smulders F.J.M. (2017). The effects of atmospheric pressure cold plasma treatment on microbiological, physical-chemical and sensory characteristics of vacuum packaged beef loin. Meat Sci..

[b0085] Bermúdez-Aguirre D., Wemlinger E., Pedrow P., Barbosa-Cánovas G., Garcia-Perez M. (2013). Effect of atmospheric pressure cold plasma (APCP) on the inactivation of Escherichia coli in fresh produce. Food Control.

[b0090] Beyrer M., Pina-Perez M.C., Martinet D., Andlauer W. (2020). Cold plasma processing of powdered Spirulina algae for spore inactivation and preservation of bioactive compounds. Food Control.

[b0095] Bian X., Chen J.R., Yang Y., Yu D.H., Ma Z.Q., Ren L.K., Zhang N. (2021). Effects of fermentation on the structure and physical properties of glutinous proso millet starch. Food Hydrocoll..

[b0100] Bithell, R.M., 1982. Package and sterilizing process for same. US Patent No. 4,321,232. U.S. Patent and Trademark Office, Washington, DC.

[b0105] Bompeix, G., Sardo, A., 1999. Process for the treatment of fruits and vegetables. U.S. Patent No. 5,858,436. U.S. Patent and Trademark Office, Washington, DC.

[b0110] Boucher, R.M.G., 1980. Seededgas plasma sterilization method. US Patent No. 4,207,286. U.S. Patent and Trademark Office, Washington, DC.

[b0115] Bußler S., Ehlbeck J., Schlüter O.K. (2017). Pre-drying treatment of plant related tissues using plasma processed air: Impact on enzyme activity and quality attributes of cut apple and potato. Innovative Food Sci. Emerg. Technol..

[b0120] Butscher D., Van Loon H., Waskow A., von Rohr P.R., Schuppler M. (2016). Plasma inactivation of microorganisms on sprout seeds in a dielectric barrier discharge. Int. J. Food Microbiol..

[b0125] Cahill O.J., Claro T., Cafolla A.A., Stevens N.T., Daniels S., Humphreys H. (2017). Decontamination of hospital surfaces with multijet cold plasma: A method to enhance infection prevention and control?. Infect. Control Hosp. Epidemiol..

[b0130] Chaple S., Sarangapani C., Jones J., Carey E., Causeret L., Genson A., Bourke P. (2020). Effect of atmospheric cold plasma on the functional properties of whole wheat (*Triticum aestivum* L.) grain and wheat flour. Innovative Food Sci. Emerg. Technol..

[b0135] Chen C., Liu C., Jiang A., Guan Q., Sun X., Liu S., Hu W. (2019). The effects of cold plasma-activated water treatment on the microbial growth and antioxidant properties of fresh-cut pears. Food Bioprocess Technol..

[b0140] Chen D., Peng P., Zhou N., Cheng Y., Min M., Ma Y., Ruan R. (2019). Evaluation of *Cronobacter sakazakii* inactivation and physicochemical property changes of non-fat dry milk powder by cold atmospheric plasma. Food Chem..

[b0145] Chen J., Wang S.Z., Chen J.Y., Chen D.Z., Deng S.G., Xu B. (2019). Effect of cold plasma on maintaining the quality of chub mackerel (*Scomber japonicus*): biochemical and sensory attributes. J. Sci. Food Agric..

[b0150] Chizoba Ekezie F.G., Sun D.W., Han Z., Cheng J.H. (2017). Microwave-assisted food processing technologies for enhancing product quality and process efficiency: A review of recent developments. Trends Food Sci. Technol..

[b0155] Chizoba Ekezie F.-G., Sun D.-W., Cheng J.-H. (2017). A review on recent advances in cold plasma technology for the food industry: Current applications and future trends. Trends Food Sci. Technol..

[b0160] Chutia H., Mahanta C.L. (2021). Influence of cold plasma voltage and time on quality attributes of tender coconut water (*Cocos nucifera* L.) and degradation kinetics of its blended beverage. J. Food Process. Preserv..

[b0165] Claro T., Cahill O.J., O’Connor N., Daniels S., Humphreys H. (2015). Cold-air atmospheric pressure plasma against *Clostridium difficile* spores: A potential alternative for the decontamination of hospital inanimate surfaces. Infect. Control Hosp. Epidemiol..

[b0170] Coutinho N.M., Silveira M.R., Fernandes L.M., Moraes J., Pimentel T.C., Freitas M.Q., Cruz A.G. (2019). Processing chocolate milk drink by low-pressure cold plasma technology. Food Chem..

[b0175] Coutinho N.M., Silveira M.R., Pimentel T.C., Freitas M.Q., Moraes J., Fernandes L.M., Cruz A.G. (2019). Chocolate milk drink processed by cold plasma technology: Physical characteristics, thermal behavior and microstructure. LWT - Food Sci. Technol..

[b0180] Coutinho N.M., Silveira M.R., Rocha R.S., Moraes J., Ferreira M.V.S., Pimentel T.C., Freitas M.Q., Silva M.C., Raices R.S., Ranadheera C.S., Cruz A.G. (2018). Cold plasma processing of milk and dairy products. Trends Food Sci. Technol..

[b0185] Cui H., Bai M., Yuan L., Surendhiran D., Lin L. (2018). Sequential effect of phages and cold nitrogen plasma against *Escherichia coli* O157:H7 biofilms on different vegetables. Int. J. Food Microbiol..

[b0190] Cui H., Yang X., Abdel-Samie M.A., Lin L. (2020). Cold plasma treated phlorotannin/Momordica charantia polysaccharide nanofiber for active food packaging. Carbohydr. Polym..

[b0195] Cullen P.J., Lalor J., Scally L., Boehm D., Milosavljević V., Bourke P., Keener K. (2018). Translation of plasma technology from the lab to the food industry. Plasma Processes Polym..

[b0200] Dantas A.M., Batista J.D.F., dos Santos Lima M., Fernandes F.A., Rodrigues S., Magnani M., Borges G.D.S.C. (2021). Effect of cold plasma on açai pulp: enzymatic activity, color and bioaccessibility of phenolic compounds. LWT - Food Sci. Technol..

[b0205] Dasan B.G., Boyaci I.H. (2017). Effect of cold atmospheric plasma on inactivation of *escherichia coli* and physicochemical properties of apple, orange, tomato juices, and sour cherry nectar. Food Bioprocess Technol..

[b0210] Dasan B.G., Boyaci I.H., Mutlu M. (2017). Nonthermal plasma treatment of *Aspergillus* spp. spores on hazelnuts in an atmospheric pressure fluidized bed plasma system: Impact of process parameters and surveillance of the residual viability of spores. J. Food Eng..

[b0215] Dasan B.G., Yildirim T., Boyaci I.H. (2018). Surface decontamination of eggshells by using non-thermal atmospheric plasma. Int. J. Food Microbiol..

[b0220] de Castro D.R.G., Mar J.M., da Silva L.S., da Silva K.A., Sanches E.A., de Araújo Bezerra J., Campelo P.H. (2020). Dielectric barrier atmospheric cold plasma applied on camu-camu juice processing: Effect of the excitation frequency. Food Res. Int..

[b0225] de Souza Silva D.A., da Silva Campelo M.C., de Oliveira Soares RebouçAs L.U.C.A.S., de Oliveira Vitoriano J., Alves C., Alves da Silva J.B., de Oliveira Lima P. (2019). Use of cold atmospheric plasma to preserve the quality of white shrimp (Litopenaeus vannamei). J. Food Prot..

[b0230] Deng L.Z., Mujumdar A.S., Pan Z., Vidyarthi S.K., Xu J., Zielinska M., Xiao H.W. (2020). Emerging chemical and physical disinfection technologies of fruits and vegetables: a comprehensive review. Crit. Rev. Food Sci. Nutr..

[b0235] Dinika I., Verma D.K., Balia R., Utama G.L., Patel A. (2020). Potential of cheese whey bioactive peptides in the development of antimicrobial edible film composite: A review of recent trends. Trends Food Sci. Technol..

[b9115] Devi Y., Thirumdas R., Sarangapani C., Deshmukh R.R., Annapure U.S. (2017). Influence of cold plasma on fungal growth and aflatoxins production on groundnuts. Food Control.

[b0240] Ekezie F.G.C., Sun D.W., Cheng J.H. (2017). A review on recent advances in cold plasma technology for the food industry: Current applications and future trends. Trends Food Sci. Technol..

[b0245] Ermolaeva S.A., Varfolomeev A.F., Chernukha M.Y., Yurov D.S., Vasiliev M.M., Kaminskaya A.A., Moisenovich M.M., Romanova J.M., Murashev A.N., Selezneva I.I., Shimizu T. (2011). Bactericidal effects of non-thermal argon plasma *in vitro*, in biofilms and in the animal model of infected wounds. J. Med. Microbiol..

[b0250] Fai A.E.C., Alves de Souza M.R., de Barros S.T., Bruno N.V., Ferreira M.S.L., Gonçalves T.C.B.D.A., Branco de Andrade É.C. (2016). Development and evaluation of biodegradable films and coatings obtained from fruit and vegetable residues applied to fresh-cut carrot (*Daucus carota* L.). Postharvest Biol. Technol..

[b0255] Feizollahi E., Misra N.N., Roopesh M.S. (2021). Factors influencing the antimicrobial efficacy of Dielectric Barrier Discharge (DBD) Atmospheric Cold Plasma (ACP) in food processing applications. Crit. Rev. Food Sci. Nutr..

[b0260] Fernandes F.A., Santos V.O., Rodrigues S. (2019). Effects of glow plasma technology on some bioactive compounds of acerola juice. Food Res. Int..

[b0265] Fernández A., Noriega E., Thompson A. (2013). Inactivation of *Salmonella enterica* serovar *Typhimurium* on fresh produce by cold atmospheric gas plasma technology. Food Microbiol..

[b9030] Fröhling A., Durek J., Schnabel U., Ehlbeck J., Bolling J., Schlüter O. (2012). Indirect plasma treatment of fresh pork: Decontamination efficiency and effects on quality attributes. Innovative Food Science & Emerging Technologies.

[b0270] Gandhi N., Singh B., Singh P., Sharma S. (2021). Functional, Rheological, Morphological, and Micro-Structural Properties of Extrusion-Processed Corn and Potato Starches. Starch-Stärke.

[b0275] Ganiari S., Choulitoudi E., Oreopoulou V. (2017). Edible and active films and coatings as carriers of natural antioxidants for lipid food. Trends Food Sci. Technol..

[b0280] Gavahian M., Chu Y.H., Khaneghah A.M., Barba F.J., Misra N.N. (2018). A critical analysis of the cold plasma induced lipid oxidation in foods. Trends Food Sci. Technol..

[b0285] Georgescu N., Apostol L., Gherendi F. (2017). Inactivation of *Salmonella enterica* serovar *Typhimurium* on egg surface, by direct and indirect treatments with cold atmospheric plasma. Food Control.

[b0290] Gu Y., Shi W., Liu R., Xing Y., Yu X., Jiang H. (2021). Cold plasma enzyme inactivation on dielectric properties and freshness quality in bananas. Innovative Food Sci. Emerg. Technol..

[b0295] Han L., Boehm D., Amias E., Milosavljević V., Cullen P.J., Bourke P. (2016). Atmospheric cold plasma interactions with modified atmosphere packaging inducer gases for safe food preservation. Innovative Food Sci. Emerg. Technol..

[b0300] Han L., Patil S., Boehm D., Milosavljević V., Cullen P.J., Bourke P. (2016). Mechanisms of inactivation by high-voltage atmospheric cold plasma differ for Escherichia coli and Staphylococcus aureus. Appl. Environ. Microbiol..

[b0305] Han S.H., Suh H.J., Hong K.B., Kim S.Y., Min S.C. (2016). Oral Toxicity of Cold Plasma-Treated Edible Films for Food Coating. J. Food Sci..

[b0310] Hatab S. (2018). Effect of cold atmospheric plasma (CAP) on endogenous enzyme activity and quality parameters of hairtail *(Trichiurus japonicu*s). J. Food Dairy Sci..

[b0315] Heidemann H.M., Dotto M.E., Laurindo J.B., Carciofi B.A., Costa C. (2019). Cold plasma treatment to improve the adhesion of cassava starch films onto PCL and PLA surface. Colloids Surf., A.

[b0320] Herianto S., Hou C.Y., Lin C.M., Chen H.L. (2021). Nonthermal plasma-activated water: A comprehensive review of this new tool for enhanced food safety and quality. Compr. Rev. Food Sci. Food Saf..

[b0325] Herrmann, H.W., Selwyn, G.S., 2001. Atmospheric-pressure plasma decontamination/sterilization chamber. U.S. Patent No. 6,228,330. U.S. Patent and Trademark Office, Washington, DC.

[b0330] Hertwig C., Leslie A., Meneses N., Reineke K., Rauh C., Schlüter O. (2017). Inactivation of *Salmonella enteritidis* PT30 on the surface of unpeeled almonds by cold plasma. Innovative Food Sci. Emerg. Technol..

[b0335] Hertwig C., Reineke K., Ehlbeck J., Knorr D., Schlüter O. (2015). Decontamination of whole black pepper using different cold atmospheric pressure plasma applications. Food Control.

[b0340] Hertwig C., Reineke K., Rauh C., Schlüter O. (2017). Factors involved in *Bacillus* spore’s resistance to cold atmospheric pressure plasma. Innovative Food Sci. Emerg. Technol..

[b0345] Hosseini S.M., Hosseinzadeh Samani B., Rostami S., Lorigooini Z. (2021). Design and characterisation of jet cold atmospheric pressure plasma and its effect on *Escherichia coli*, colour, pH, and bioactive compounds of sour cherry juice. Int. J. Food Sci. Technol..

[b0350] Hou Y., Wang R., Gan Z., Shao T., Zhang X., He M., Sun A. (2019). Effect of cold plasma on blueberry juice quality. Food Chem..

[b0355] Hury S., Vidal D.R., Desor F., Pelletier J., Lagarde T. (1998). A parametric study of the destruction efficiency of *Bacillus* spores in low pressure oxygen-based plasmas. Lett. Appl. Microbiol..

[b0360] Iqbal A., Murtaza A., Hu W., Ahmad I., Ahmed A., Xu X. (2019). Activation and inactivation mechanisms of polyphenol oxidase during thermal and non-thermal methods of food processing. Food Bioprod. Process..

[b0365] Issaoui M., Delgado A.M., Caruso G., Micali M., Barbera M., Atrous H., Chammem N. (2020). Phenols, flavors, and the mediterranean diet. J. AOAC Int..

[b0370] Iulietto M.F., Sechi P., Borgogni E., Cenci-Goga B.T. (2015). Meat spoilage: A critical review of a neglected alteration due to ropy slime producing bacteria. Ital. J. Anim. Sci..

[b0375] Jacobs, P.T., Lin, S.M., 1987. Hydrogen, US Patent No. 4,643,786. U.S. Patent and Trademark Office, Washington, DC.

[b0380] Jacofsky, M.C., Watson, G.A., 2016. Method and apparatus for cold plasma food contact surface sanitation. U.S. Patent No. 9,295,280. U.S. Patent and Trademark Office, Washington, DC.

[b0385] Kang J.H., Bai J., Min S.C. (2021). Inactivation of Indigenous Microorganisms and Salmonella in Korean Rice Cakes by In-Package Cold Plasma Treatment. Int. J. Environ. Res. Public Health.

[b0390] Karami-Gadallo L., Ghoranneviss M., Ataie-Fashtami L., Pouladian M., Sardari D. (2017). Enhancement of cancerous cells treatment by applying cold atmospheric plasma and photo dynamic therapy simultaneously. Clin. Plasm. Med..

[b0395] Kashfi A.S., Ramezan Y., Khani M.R. (2020). Simultaneous study of the antioxidant activity, microbial decontamination and color of dried peppermint (*Mentha piperita* L.) using low pressure cold plasma. LWT - Food Sci. Technol..

[b0400] Khadtare S., Bansode A.S., Mathe V.L., Shrestha N.K., Bathula C., Han S.H., Pathan H.M. (2017). Effect of oxygen plasma treatment on performance of ZnO based dye sensitized solar cells. J. Alloy. Compd..

[b0405] Khamsen N., Akkarachanchainon A., Fookiat K., Srisala J., Chomchuen S., Kanokbannakorn W., Srisonphan S. (2016). Atmospheric cold plasma via fringe field enhanced corona discharge on single dielectric barrier for large-volume applications. Procedia Comput. Sci..

[b0410] Khamsen N., Akkarachanchainon A., Teerakawanich N., Srisonphan S. (2016). Organic and bio material surface modification via corona discharge induced atmospheric-cold plasma. Procedia Comput. Sci..

[b0415] Khan M.K., Ahmad K., Hassan S., Imran M., Ahmad N., Xu C. (2018). Effect of novel technologies on polyphenols during food processing. Innovative Food Sci. Emerg. Technol..

[b0420] Khatun A., Waters D.L., Liu L. (2019). A review of rice starch digestibility: effect of composition and heat-moisture processing. Starch-Stärke.

[b0425] Kim H.J., Yong H.I., Park S., Kim K., Choe W., Jo C. (2015). Microbial safety and quality attributes of milk following treatment with atmospheric pressure encapsulated dielectric barrier discharge plasma. Food Control.

[b0430] Kim J.H., Min S.C. (2017). Microwave-powered cold plasma treatment for improving microbiological safety of cherry tomato against *Salmonella*. Postharvest Biol. Technol..

[b0435] Kim J.H., Min S.C. (2018). Moisture vaporization-combined helium dielectric barrier discharge-cold plasma treatment for microbial decontamination of onion flakes. Food Control.

[b9105] Kim B., Yun H., Jung S., Jung Y., Jung H., Choe W., Jo C. (2011). Effect of atmospheric pressure plasma on inactivation of pathogens inoculated onto bacon using two different gas compositions. Food microbiology.

[b0440] Kim J.S., Lee E.J., Kim Y.J. (2014). Inactivation of Campylobacter jejuni with dielectric barrier discharge plasma using air and nitrogen gases. Foodborne Pathog. Dis..

[b0445] Klämpfl T.G., Isbary G., Shimizu T., Li Y.F., Zimmermann J.L., Stolz W., Schlegel J., Morfill G.E., Schmidt H.U. (2012). Cold atmospheric air plasma sterilization against spores and other microorganisms of clinical interest. Appl. Environ. Microbiol..

[b0450] Koddy J.K., Miao W., Hatab S., Tang L., Xu H., Nyaisaba B.M., Deng S. (2021). Understanding the role of atmospheric cold plasma (ACP) in maintaining the quality of hairtail (Trichiurus Lepturus). Food Chem..

[b0455] Korachi M., Ozen F., Aslan N., Vannini L., Guerzoni M.E., Gottardi D., Ekinci F.Y. (2015). Biochemical changes to milk following treatment by a novel, cold atmospheric plasma system. Int. Dairy J..

[b0460] Kovalova Z., Leroy M., Kirkpatrick M.J., Odic E., Machala Z. (2016). Corona discharges with water electrospray for *Escherichia coli* biofilm eradication on a surface. Bioelectrochemistry.

[b0465] Kumar R., Singh P., Kumar S., Pereira M.L., Silva D., Salisu, Tahir M. (2017). Plasma treatment – A tool to improve seed quality – A review. Adv. Res..

[b0470] Kumari M., Mahajan H., Joshi R., Gupta M. (2017). Development and structural characterization of edible films for improving fruit quality. Food Packag. Shelf Life.

[b0475] Lacombe A., Niemira B.A., Gurtler J.B., Fan X., Sites J., Boyd G., Chen H. (2015). Atmospheric cold plasma inactivation of aerobic microorganisms on blueberries and effects on quality attributes. Food Microbiol..

[b0480] Lacombe A., Niemira B.A., Gurtler J.B., Sites J., Boyd G., Kingsley D.H., Chen H. (2017). Nonthermal inactivation of norovirus surrogates on blueberries using atmospheric cold plasma. Food Microbiol..

[b0485] Langmuir I. (1928). Oscillations in ionized gases. *Phy.: I. Langmuir*.

[b0490] Laroussi, M., 1999. Sterilization of liquids using a plasma glow discharge. US Patent No. 5,876,663.

[b0495] Lehmann A., Pietag F., Arnold T. (2017). Human health risk evaluation of a microwave-driven atmospheric plasma jet as medical device. Clin. Plasma Med..

[b0500] Leite A.K., Fonteles T.V., Miguel T.B., da Silva G.S., de Brito E.S., Alves Filho E.G., Rodrigues S. (2021). Atmospheric cold plasma frequency imparts changes on cashew apple juice composition and improves vitamin C bioaccessibility. Food Res. Int..

[b0505] Li X., Li M., Ji N., Jin P., Zhang J., Zheng Y., Li F. (2019). Cold plasma treatment induces phenolic accumulation and enhances antioxidant activity in fresh-cut pitaya *(Hylocereus undatu*s) fruit. LWT - Food Sci. Technol..

[b0510] Liang Y., Wu Y., Sun K., Chen Q., Shen F., Zhang J., Fang J. (2012). Rapid inactivation of biological species in the air using atmospheric pressure nonthermal plasma. Environ. Sci. Technol..

[b0515] Liao X., Li J., Muhammad A.I., Suo Y., Chen S., Ye X., Ding T. (2018). Application of a dielectric barrier discharge atmospheric cold plasma (Dbd-Acp) for *Eshcerichia coli* inactivation in apple juice. J. Food Sci..

[b0520] Liao X., Liu D., Xiang Q., Ahn J., Chen S., Ye X., Ding T. (2017). Inactivation mechanisms of non-thermal plasma on microbes: A review. Food Control.

[b0525] Liao X., Xiang Q., Liu D., Chen S., Ye X., Ding T. (2017). Lethal and sublethal effect of a dielectric barrier discharge atmospheric cold plasma on *Staphylococcus aureus*. J. Food Prot..

[b0530] Liu C.T., Kumakura T., Ishikawa K., Hashizume H., Takeda K., Ito M., Wu J.S. (2016). Effects of assisted magnetic field to an atmospheric-pressure plasma jet on radical generation at the plasma-surface interface and bactericidal function. Plasma Sources Sci. Technol..

[b0535] Liu L., Li S., Zheng J., Bu T., He G., Wu J. (2020). Safety considerations on food protein-derived bioactive peptides. Trends Food Sci. Technol..

[b0540] Liu Z., Zhao W., Zhang Q., Gao G., Meng Y. (2021). Effect of cold plasma treatment on sterilizing rate and quality of kiwi turbid juice. J. Food Process Eng.

[b0545] Los A., Ziuzina D., Akkermans S., Boehm D., Cullen P.J., Van Impe J., Bourke P. (2018). Improving microbiological safety and quality characteristics of wheat and barley by high voltage atmospheric cold plasma closed processing. Food Res. Int..

[b0550] Lunov O., Zablotskii V., Churpita O., Jäger A., Polívka L., Syková E., Kubinová Š. (2016). The interplay between biological and physical scenarios of bacterial death induced by non-thermal plasma. Biomaterials.

[b0555] Ma L., Zhang M., Bhandari B., Gao Z. (2017). Recent developments in novel shelf life extension technologies of fresh-cut fruits and vegetables. Trends Food Sci. Technol..

[b0560] Machado-Velasco K.M., Vélez-Ruiz J.F. (2008). Study of physical properties in mexican foods during freezing and frozen storage. Revista Mexicana De Ingeniería Química.

[b0565] Mahnot N.K., Siyu L.P., Wan Z., Keener K.M., Misra N.N. (2020). In-package cold plasma decontamination of fresh-cut carrots: microbial and quality aspects. J. Phys. D Appl. Phys..

[b0570] Mandal R., Singh A., Singh A.P. (2018). Recent developments in cold plasma decontamination technology in the food industry. Trends Food Sci. Technol..

[b0575] Marquez R., Di Pierro P., Mariniello L., Esposito M., Giosafatto C., Porta R. (2017). Fresh-cut fruit and vegetable coatings by transglutaminase-crosslinked whey protein/pectin edible films. LWT - Food Sci. Technol..

[b0580] Menashi, W.P., 1968. Treatment of surfaces. US Patent No. 3,383,163. U.S. Patent and Trademark Office, Washington, DC.

[b0585] Metelmann H.R., Seebauer C., Miller V., Fridman A., Bauer G., Graves D.B., von Woedtke T. (2018). Clinical experience with cold plasma in the treatment of locally advanced head and neck cancer. Clin. Plas. Med..

[b0590] Min S.C., Roh S.H., Niemira B.A., Boyd G., Sites J.E., Fan X., Sokorai K., Jin T.Z. (2018). In-package atmospheric cold plasma treatment of bulk grape tomatoes for microbiological safety and preservation. Food Res. Int..

[b0595] Min S., Roh S.H., Niemira B.A., Sites J.E., Boyd G., Lacombe A. (2016). Dielectric barrier discharge atmospheric cold plasma inhibits *Escherichia coli* O157:H7, *Salmonella*, *Listeria monocytogenes*, and Tulane virus in Romaine lettuce. Int. J. Food Microbiol..

[b0600] Min S., Roh S., Niemira B., Boyd G., Sites J., Uknalis J., Fan X. (2017). In-package inhibition of *E. coli* O157:H7 on bulk Romaine lettuce using cold plasma. Food Microbiol..

[b0605] Mir S.A., Shah M.A., Mir M.M. (2016). Understanding the role of plasma technology in food industry. Food Bioprocess Technol..

[b0610] Mishra R., Bhatia S., Pal R., Visen A., Trivedi H. (2016). Cold plasma: Emerging as the new standard in food safety. Int. J. f Eng. Sci..

[b0615] Misra N.N., Jo C. (2017). Applications of cold plasma technology for microbiological safety in meat industry. Trends Food Sci. Technol..

[b0620] Misra N.N., Keener K.M., Bourke P., Mosnier J.P., Cullen P.J. (2014). In-package atmospheric pressure cold plasma treatment of cherry tomatoes. J. Biosci. Bioeng..

[b0625] Misra N.N., Pankaj S.K., Segat A., Ishikawa K. (2016). Cold plasma interactions with enzymes in foods and model systems. Trends Food Sci. Technol..

[b0630] Misra N.N., Tiwari B.K., Raghavarao K.S.M.S., Cullen P.J. (2011). Nonthermal plasma inactivation of food-borne pathogens. Food Eng. Rev..

[b0635] Misra N.N., Yadav B., Roopesh M.S., Jo C. (2019). Cold plasma for effective fungal and mycotoxin control in foods: mechanisms, inactivation effects, and applications. Compr. Rev. Food Sci. Food Saf..

[b0640] Mohammadpour H., Zarei M., Cullen P.J., Valtchev P., Schindeler A., Dehghani F. (2021). Potential application of non-thermal atmospheric plasma in reducing the activity of Pseudomonas-secreted proteases in milk. Int. Dairy J..

[b0645] Mollakhalili-Meybodi N., Yousefi M., Nematollahi A., Khorshidian N. (2021). Effect of atmospheric cold plasma treatment on technological and nutrition functionality of protein in foods. Eur. Food Res. Technol..

[b0650] Moosavi M.H., Khani M.R., Shokri B., Hosseini S.M., Shojaee-Aliabadi S., Mirmoghtadaie L. (2020). Modifications of protein-based films using cold plasma. Int. J. Biol. Macromol..

[b0655] Mošovská S., Medvecká V., Halászová N., Ďurina P., Valík Ľ., Mikulajová A., Zahoranová A. (2018). Cold atmospheric pressure ambient air plasma inhibition of pathogenic bacteria on the surface of black pepper. Food Res. Int..

[b0660] Moutiq R., Misra N.N., Mendonca A., Keener K. (2020). In-package decontamination of chicken breast using cold plasma technology: Microbial, quality and storage studies. Meat Sci..

[b0665] Musavu Ndob A., Lebert A. (2018). Prediction of pH and aw of pork meat by a thermodynamic model: New developments. Meat Sci..

[b0670] Mustafa M., Fu X., Liu Y., Abbas Y., Wang H., Lu W. (2018). Volatile organic compounds (VOCs) removal in non-thermal plasma double dielectric barrier discharge reactor. J. Hazard. Mater..

[b0675] Nelson C.L., Berger T.J. (1989). Inactivation of microorganisms by oxygen gas plasma. Curr. Microbiol..

[b0680] Ng S.W., Lu P., Rulikowska A., Boehm D., O'Neill G., Bourke P. (2021). The effect of atmospheric cold plasma treatment on the antigenic properties of bovine milk casein and whey proteins. Food Chem..

[b0685] Nyaisaba B.M., Miao W., Hatab S., Siloam A., Chen M., Deng S. (2019). Effects of cold atmospheric plasma on squid proteases and gel properties of protein concentrate from squid (*Argentinus ilex*) mantle. Food Chem..

[b0690] O’Connor N., Cahill O., Daniels S., Galvin S., Humphreys H. (2014). Cold atmospheric pressure plasma and decontamination. Can it contribute to preventing hospital-acquired infections?. J. Hosp. Infect..

[b0695] Odeyemi O.A., Alegbeleye O.O., Strateva M., Stratev D. (2020). Understanding spoilage microbial community and spoilage mechanisms in foods of animal origin. Compr. Rev. Food Sci. Food Saf..

[b0700] Olatunde O.O., Benjakul S. (2018). Nonthermal processes for shelf-life extension of seafoods: A revisit. Compr. Rev. Food Sci. Food Saf..

[b0705] Olatunde O.O., Benjakul S. (2020). Antioxidants from crustaceans: A panacea for lipid oxidation in marine-based foods. Food Rev. Int..

[b0710] Olatunde O.O., Benjakul S., Vongkamjan K. (2019). Dielectric barrier discharge high voltage cold atmospheric plasma: An innovative nonthermal technology for extending the shelf-life of Asian sea bass slices. J. Food Sci..

[b0715] Olatunde O.O., Benjakul S., Vongkamjan K. (2020). Microbial diversity, shelf-life and sensory properties of Asian sea bass slices with combined treatment of liposomal encapsulated ethanolic coconut husk extract and high voltage cold plasma. LWT - Food Sci. Technol..

[b0720] Olatunde O.O., Benjakul S., Vongkamjan K. (2020). Shelf-life of refrigerated Asian sea bass slices treated with cold plasma as affected by gas composition in packaging. Int. J. Food Microbiol..

[b0725] Olatunde O.O., Chantakun K., Benjakul S. (2021). Microbial, chemical qualities and shelf-life of blue swimming crab (Portunus armatus) lump meat as influenced by in-package high voltage cold plasma treatment. Food Biosci..

[b0730] Olatunde O.O., Shiekh K.A., Benjakul S. (2021). Pros and cons of cold plasma technology as an alternative non-thermal processing technology in seafood industry. Trends Food Sci. Technol..

[b0735] Olschewski E.S. (2011). Aplicaciones de la física de plasmas en la industria. Ingen. Ind..

[b0740] Paixão L.M., Fonteles T.V., Oliveira V.S., Fernandes F.A., Rodrigues S. (2019). Cold plasma effects on functional compounds of siriguela juice. Food Bioprocess Technol..

[b0745] Pankaj S.K., Keener K.M. (2017). Cold plasma: Background, applications and current trends. Curr. Opin. Food Sci..

[b0750] Pankaj S.K., Wan Z., Keener K.M. (2018). Effects of cold plasma on food quality: A review. Foods.

[b0755] Pankaj S.K., Wan Z., Colonna W., Keener K.M. (2017). Effect of high voltage atmospheric cold plasma on white grape juice quality. J. Sci. Food Agric..

[b0760] Panpipat W., Chaijan M. (2020). Effect of atmospheric pressure cold plasma on biophysical properties and aggregation of natural actomyosin from threadfin bream (Nemipterus bleekeri). Food Bioprocess Technol..

[b0765] Park S.Y., Ha S. Do. (2018). Assessment of cold oxygen plasma technology for the inactivation of major foodborne viruses on stainless steel. J. Food Eng..

[b0770] Paskalov, G., 2013. Sterilization using plasma generated NOx. U.S. Patent Application No. 13/712,373. U.S. Patent and Trademark Office, Washington, DC.

[b0775] Paskalov, G., 2014. Plasma powder sterilization apparatus and methods.U.S. Patent No. 8,771,595. U.S. Patent and Trademark Office, Washington, DC.

[b0780] Pasquali F., Stratakos A.C., Koidis A., Berardinelli A., Cevoli C., Ragni L., Trevisani M. (2016). Atmospheric cold plasma process for vegetable leaf decontamination: A feasibility study on radicchio (red chicory, *Cichorium intybus* L.). Food Control.

[b0785] Patil S., Moiseev T., Misra N.N., Cullen P.J., Mosnier J.P., Keener K.M., Bourke P. (2014). Influence of high voltage atmospheric cold plasma process parameters and role of relative humidity on inactivation of *Bacillus atrophaeus* spores inside a sealed package. J. Hosp. Infect..

[b0790] Pereira A.G., Fraga-Corral M., García-Oliveira P., Jimenez-Lopez C., Lourenço-Lopes C., Carpena M., Simal-Gandara J. (2020). Culinary and nutritional value of edible wild plants from northern Spain rich in phenolic compounds with potential health benefits. Food Funct..

[b0795] Ponraj S.B., Sharp J.A., Kanwar J.R., Sinclair A.J., Kviz L., Nicholas K.R., Dai X.J. (2017). Argon gas plasma to decontaminate and extend shelf life of milk. Plasma Processes Polym..

[b0800] Porto E., Alves Filho E.G., Silva L.M.A., Fonteles T.V., do Nascimento R.B.R., Fernandes F.A. (2020). Ozone and plasma processing effect on green coconut water. Food Res. Int..

[b0805] Prasad P., Mehta D., Bansal V., Sangwan R.S. (2017). Effect of atmospheric cold plasma (ACP) with its extended storage on the inactivation of *Escherichia coli* inoculated on tomato. Food Res. Int..

[b0810] Ramazzina I., Berardinelli A., Rizzi F., Tappi S., Ragni L., Sacchetti G., Rocculi P. (2015). Effect of cold plasma treatment on physico-chemical parameters and antioxidant activity of minimally processed kiwifruit. Postharvest Biol. Technol..

[b0815] Ramos B., Miller F.A., Brandão T.R.S., Teixeira P., Silva C.L.M. (2013). Fresh fruits and vegetables - An overview on applied methodologies to improve its quality and safety. Innovative Food Sci. Emerg. Technol..

[b0820] Rana S., Mehta D., Bansal V., Shivhare U.S., Yadav S.K. (2020). Atmospheric cold plasma (ACP) treatment improved in-package shelf-life of strawberry fruit. J. Food Sci. Technol..

[b0825] Rathod N.B., Kahar S.P., Ranveer R.C., Annapure U.S. (2021). Cold plasma an emerging nonthermal technology for milk and milk products: A review. Int. J. Dairy Technol..

[b0830] Rathod N.B., Ranveer R.C., Bhagwat P.K., Ozogul F., Benjakul S., Pillai S., Annapure U.S. (2021). Cold plasma for the preservation of aquatic food products: An overview. Compr. Rev. Food Sci. Food Saf..

[b0835] Ribeiro G.P., Villas-Bôas J.K., Spinosa W.A., Prudencio S.H. (2018). Influence of freezing, pasteurization and maturation on Tiúba honey quality. LWT - Food Sci. Technol..

[b0840] Ritter A.C., Santi L., Vannini L., Beys-da-Silva W.O., Gozzi G., Yates J., Brandelli A. (2018). Comparative proteomic analysis of foodborne *Salmonella enteritidis* SE86 subjected to cold plasma treatment. Food Microbiol..

[b9010] Roh S.H., Lee S.Y., Park H.H., Lee E.S., Min S.C. (2019). Effects of the treatment parameters on the efficacy of the inactivation of Salmonella contaminating boiled chicken breast by in-package atmospheric cold plasma treatment. International Journal of Food Microbiology.

[b0845] Romani V.P., Olsen B., Collares M.P., Oliveira J.R.M., Prentice C., Martins V.G. (2020). Cold plasma and carnauba wax as strategies to produce improved bi-layer films for sustainable food packaging. Food Hydrocoll..

[b0850] Rothrock M.J., Zhuang H., Lawrence K.C., Bowker B.C., Gamble G.R., Hiett K.L. (2017). In-Package inactivation of pathogenic and spoilage bacteria associated with poultry using dielectric barrier discharge-cold plasma treatments. Curr. Microbiol..

[b0855] Roy N.C., Hasan M.M., Kabir A.H., Reza M.A., Talukder M.R., Chowdhury A.N. (2018). Atmospheric pressure gliding arc discharge plasma treatments for improving germination, growth and yield of wheat. Plasma Sci. Technol.

[b0860] Royintarat T., Seesuriyachan P., Boonyawan D., Choi E.H., Wattanutchariya W. (2019). Mechanism and optimization of non-thermal plasma-activated water for bacterial inactivation by underwater plasma jet and delivery of reactive species underwater by cylindrical DBD plasma. Curr. Appl Phys..

[b0865] Rozenberg S., Body J.J., Bruyere O., Bergmann P., Brandi M.L., Cooper C., Reginster J.Y. (2016). Effects of dairy products consumption on health: benefits and beliefs—a commentary from the Belgian Bone Club and the European Society for Clinical and Economic Aspects of Osteoporosis, Osteoarthritis and Musculoskeletal Diseases. Calcif. Tissue Int..

[b0870] Santos L.C.O., Cubas A.L.V., Moecke E.H.S., Ribeiro D.H.B., Amante E.R. (2018). Use of cold plasma to inactivate *Escherichia coli* and physicochemical evaluation in pumpkin puree. J. Food Prot..

[b0875] Sarangapani C., O’Toole G., Cullen P.J., Bourke P. (2016). Atmospheric cold plasma dissipation efficiency of agrochemicals on blueberries. Innovative Food Sci. Emerg. Technol..

[b9020] Sarangapani C., Keogh D.R., Dunne J., Bourke P., Cullen P.J. (2017). Characterisation of cold plasma treated beef and dairy lipids using spectroscopic and chromatographic methods. Food Chemistry.

[b0880] Sarangapani C., Patange A., Bourke P., Keener K., Cullen P.J. (2018). Recent advances in the application of cold plasma technology in foods. Ann. Rev. Food Sci. Technol..

[b0885] Schnabel U., Niquet R., Schlüter O., Gniffke H., Ehlbeck J. (2015). Decontamination and sensory properties of microbiologically contaminated fresh fruits and vegetables by Microwave Plasma Processed Air (PPA). J. Food Process. Preserv..

[b0890] Secci G., Parisi G. (2016). From farm to fork: Lipid oxidation in fish products. A review. Ital. J. Anim. Sci..

[b0895] Segat A., Misra N.N., Cullen P.J., Innocente N. (2015). Atmospheric pressure cold plasma (ACP) treatment of whey protein isolate model solution. Innovative Food Sci. Emerg. Technol..

[b0900] Segura-Ponce L.A., Reyes J.E., Troncoso-Contreras G., Valenzuela-Tapia G. (2018). Effect of low-pressure cold plasma (LPCP) on the wettability and the inactivation of *Escherichia coli* and *Listeria innocua* on fresh-cut apple (Granny Smith) skin. Food Bioprocess Technol..

[b0905] Shi H., Ileleji K., Stroshine R.L., Keener K., Jensen J.L. (2017). Reduction of aflatoxin in corn by high voltage atmospheric cold plasma. Food Bioprocess Technol..

[b0910] Shiekh K.A., Benjakul S. (2020). Effect of high voltage cold atmospheric plasma processing on the quality and shelf-life of Pacific white shrimp treated with Chamuang leaf extract. Innovative Food Sci. Emerg. Technol..

[b0915] Shiekh K.A., Zhou P., Benjakul S. (2021). Combined effects of pulsed electric field, Chamuang leaf extract and cold plasma on quality and shelf-life of *Litopenaeus vannamei*. Food Biosci..

[b0920] Sifuentes-Nieves I., Hernández-Hernández E., Neira-Velázquez G., Morales-Sánchez E., Mendez-Montealvo G., Velazquez G. (2019). Hexamethyldisiloxane cold plasma treatment and amylose content determine the structural, barrier and mechanical properties of starch-based films. Int. J. Biol. Macromol..

[b0925] Sifuentes-Nieves I., Mendez-Montealvo G., Flores-Silva P.C., Nieto-Pérez M., Neira-Velazquez G., Rodriguez-Fernandez O., Velazquez G. (2021). Dielectric barrier discharge and radio-frequency plasma effect on structural properties of starches with different amylose content. Innovative Food Sci. Emerg. Technol..

[b0930] Silveira M.R., Coutinho N.M., Rocha R.S., Moraes J., Esmerino E.A., Pimentel T.C., Cruz A.G. (2019). Guava flavored whey-beverage processed by cold plasma: Physical characteristics, thermal behavior and microstructure. Food Res. Int..

[b0935] Singh A., Benjakul S. (2020). The combined effect of squid pen chitooligosaccharides and high voltage cold atmospheric plasma on the shelf-life extension of Asian sea bass slices stored at 4 C. Innovative Food Sci. Emerg. Technol..

[b0940] Sonawane S.K., Patil S. (2020). Non-thermal plasma: An advanced technology for food industry. Food Sci. Technol. Int..

[b0945] Song A.Y., Oh Y.J., Kim J.E., Song K. Bin, Oh D.H., Min S.C. (2015). Cold plasma treatment for microbial safety and preservation of fresh lettuce. Food Sci. Biotechnol..

[b0950] Song Y., Fan X. (2020). Cold plasma enhances the efficacy of aerosolized hydrogen peroxide in reducing populations of *Salmonella typhimurium* and *Listeria innocua* on grape tomatoes, apples, cantaloupe and romaine lettuce. Food Microbiol..

[b0955] Soradech S., Nunthanid J., Limmatvapirat S., Luangtana-anan M. (2017). Utilization of shellac and gelatin composite film for coating to extend the shelf life of banana. Food Control.

[b0960] Srivastav P.P., Verma D.K., Patel A.R., Al-Hilphy A.R. (2020).

[b0965] Sruthi N.U., Josna K., Pandiselvam R., Kothakota A., Gavahian M., Khaneghah A.M. (2021). Impacts of cold plasma treatment on physicochemical, functional, bioactive, textural, and sensory attributes of food: a comprehensive review. Food Chem..

[b0970] Starek A., Pawłat J., Chudzik B., Kwiatkowski M., Terebun P., Sagan A., Andrejko D. (2019). Evaluation of selected microbial and physicochemical parameters of fresh tomato juice after cold atmospheric pressure plasma treatment during refrigerated storage. Sci. Rep..

[b0975] Stoica M., Alexe P., Mihalcea L. (2014). Atmospheric cold plasma as new strategy for foods processing - An overview. *Innovative Romanian*. Food Biotechnol..

[b0980] Sukarminah E., Djali M., Andoyo R., Mardawati E., Rialita T., Cahyana Y., Setiasih I.S. (2017). Ozonization technology and its effects on the characteristics and shelf-life of some fresh foods: A review. KnE Life Sci..

[b0985] Tappi S., Gozzi G., Vannini L., Berardinelli A., Romani S., Ragni L., Rocculi P. (2016). Cold plasma treatment for fresh-cut melon stabilization. Innovative Food Sci. Emerg. Technol..

[b0990] Tappi S., Ragni L., Tylewicz U., Romani S., Ramazzina I., Rocculi P. (2019). Browning response of fresh-cut apples of different cultivars to cold gas plasma treatment. Innovative Food Sci. Emerg. Technol..

[b0995] Thirumdas R., Deshmukh R.R., Annapure U.S. (2016). Effect of low temperature plasma on the functional properties of basmati rice flour. J. Food Sci. Technol..

[b1000] Thirumdas R., Kothakota A., Annapure U., Siliveru K., Blundell R., Gatt R., Valdramidis V.P. (2018). Plasma activated water (PAW): Chemistry, physico-chemical properties, applications in food and agriculture. Trends Food Sci. Technol..

[b1005] Thirumdas R., Sarangapani C., Annapure U.S. (2014). Cold plasma: A novel non-thermal technology for food processing. Food Biophys..

[b1010] Thirumdas R., Trimukhe A., Deshmukh R.R., Annapure U.S. (2017). Functional and rheological properties of cold plasma treated rice starch. Carbohydr. Polym..

[b1015] Thomas-Popo E., Mendonça A., Misra N.N., Little A., Wan Z., Moutiq R., Keener K. (2019). Inactivation of Shiga-toxin-producing *Escherichia coli*, *Salmonella enterica* and natural microflora on tempered wheat grains by atmospheric cold plasma. Food Control.

[b1020] Timmons C., Pai K., Jacob J., Zhang G., Ma L.M. (2018). Inactivation of *Salmonella enterica*, Shiga toxin-producing *Escherichia coli*, and *Listeria monocytogenes* by a novel surface discharge cold plasma design. Food Control.

[b1025] Tolouie H., Mohammadifar M.A., Ghomi H., Yaghoubi A.S., Hashemi M. (2018). The impact of atmospheric cold plasma treatment on inactivation of lipase and lipoxygenase of wheat germs. Innovative Food Sci. Emerg. Technol..

[b1030] Trevisani M., Berardinelli A., Cevoli C., Cecchini M., Ragni L., Pasquali F. (2017). Effects of sanitizing treatments with atmospheric cold plasma, SDS and lactic acid on verotoxin-producing *Escherichia coli* and *Listeria monocytogenes* in red chicory (radicchio). Food Control.

[b1035] Tyczkowska-Sieron E., Markiewicz J., Grzesiak B., Krukowski H., Glowacka A., Tyczkowski J. (2018). Cold atmospheric plasma inactivation of *Prototheca zopfii* isolated from bovine milk. J. Dairy Sci..

[b1040] Ucar Y., Ceylan Z., Durmus M., Tomar O., Cetinkaya T. (2021). Application of cold plasma technology in the food industry and its combination with other emerging technologies. Trends Food Sci. Technol..

[b1045] Ulbin-Figlewicz N., Brychcy E., Jarmoluk A. (2013). Effect of low-pressure cold plasma on surface microflora of meat and quality attributes. J. Food Sci. Technol..

[b1050] Verma D.K., Mahanti N.K., Thakur M., Chakraborty S.K., Srivastav P.P., Srivastav P.P., Verma D.K., Patel A.R., Al-Hilphy A.S. (2020). Emerging thermal and nonthermal technologies in food processing.

[b1055] Verma D.K., Thakur M., Kumar J., Srivastav P.P., Al-Hilphy A.R.S., Patel A., Suleria H.A.R., Srivastav P.P., Verma D.K., Patel A.R., Al-Hilphy A.S. (2020). Emerging thermal and nonthermal technologies in food processing.

[b1060] Viji P., Venkateshwarlu G., Ravishankar C.N., Gopal T.S. (2017). Role of plant extracts as natural additives in fish and fish products-A Review. Fish. Technol..

[b1065] Vlad I.E., Martin C., Toth A.R., Papp J., Anghel S.D. (2019). Bacterial inhibition effect of plasma activated water. Roman. Rep. Phys..

[b1070] Wang J.M., Zhuang H., Lawrence K., Zhang J.H. (2018). Disinfection of chicken fillets in packages with atmospheric cold plasma: effects of treatment voltage and time. J. Appl. Microbiol..

[b1075] Weltmann K.D., Kolb J.F., Holub M., Uhrlandt D., Šimek M., Ostrikov K., Hamaguchi S., Cvelbar U., Černák M., Locke B., Fridman A., Favia P., Becker K. (2019). The future for plasma science and technology. Plasma Processes Polym..

[b1080] Wohlt D., Schwarz E., Schieber A., Bader-Mittermaier S. (2021). Effects of Extraction Conditions on Banana Peel Polyphenol Oxidase Activity and Insights into Inactivation Kinetics Using Thermal and Cold Plasma Treatment. Foods.

[b1085] Won M.Y., Lee S.J., Min S.C. (2017). Mandarin preservation by microwave-powered cold plasma treatment. Innovative Food Sci. Emerg. Technol..

[b1090] Wu X., Liu Q., Luo Y., Murad M.S., Zhu L., Mu G. (2020). Improved packing performance and structure-stability of casein edible films by dielectric barrier discharges (DBD) cold plasma. Food Packag. Shelf Life.

[b1095] Wu X., Luo Y., Zhao F., Mu G. (2021). Influence of dielectric barrier discharge cold plasma on physicochemical property of milk for sterilization. Plasma Processes Polym..

[b1100] Xiang Q., Kang C., Niu L., Zhao D., Li K., Bai Y. (2018). Antibacterial activity and a membrane damage mechanism of plasma-activated water against Pseudomonas deceptionensis CM2. LWT - Food Sci. Technol..

[b1105] Xiang Q., Kang C., Zhao D., Niu L., Liu X., Bai Y. (2019). Influence of organic matters on the inactivation efficacy of plasma-activated water against *E. coli* O157: H7 and *S. aureus*. Food Control.

[b1110] Xiang Q., Liu X., Li J., Ding T., Zhang H., Zhang X., Bai Y. (2017). Influences of cold atmospheric plasma on microbial safety, physicochemical and sensorial qualities of meat products. J. Food Sci. Technol..

[b1115] Xu L., Garner A.L., Tao B., Keener K.M. (2017). Microbial inactivation and quality changes in orange juice treated by high voltage atmospheric cold plasma. Food Bioprocess Technol..

[b1120] Xu Y., Tian Y., Ma R., Liu Q., Zhang J. (2016). Effect of plasma activated water on the postharvest quality of button mushrooms, Agaricus bisporus. Food Chem..

[b1125] Yadav B., Spinelli A.C., Govindan B.N., Tsui Y.Y., McMullen L.M., Roopesh M.S. (2019). Cold plasma treatment of ready-to-eat ham: Influence of process conditions and storage on inactivation of *Listeria innocua*. Food Res. Int..

[b1130] Yagub M.T., Sen T.K., Afroze S., Ang H.M. (2014). Dye and its removal from aqueous solution by adsorption: A review. Adv. Colloid Interface Sci..

[b1135] Yaqoob M., Aggarwal P., Purandare N. (2020). Extraction of Phenolic Compounds by Supercritical Fluid Extraction.

[b1140] Yong H.I., Kim H.J., Park S., Alahakoon A.U., Kim K., Choe W., Jo C. (2015). Evaluation of pathogen inactivation on sliced cheese induced by encapsulated atmospheric pressure dielectric barrier discharge plasma. Food Microbiol..

[b1145] Yong H.I., Kim H.J., Park S., Kim K., Choe W., Yoo S.J., Jo C. (2015). Pathogen inactivation and quality changes in sliced cheddar cheese treated using flexible thin-layer dielectric barrier discharge plasma. Food Res. Int..

[b1150] Zhang M., Pang J., Bao W., Zhang W., Gao H., Wang C., Li J. (2017). Antimicrobial cotton textiles with robust superhydrophobicity via plasma for oily water separation. Appl. Surf. Sci..

[b1155] Zhang Y., Zhang J., Zhang Y., Hu H., Luo S., Zhang L., Li P. (2021). Effects of in-package atmospheric cold plasma treatment on the qualitative, metabolic and microbial stability of fresh-cut pears. J. Sci. Food Agric..

[b1160] Zhang Z., Xu Z., Cheng C., Wei J., Lan Y., Ni G., Chu P.K. (2017). Bactericidal effects of plasma induced reactive species in dielectric barrier gas–liquid discharge. Plasma Chem. Plasma Process..

[b1165] Zhao Y.M., de Alba M., Sun D.W., Tiwari B. (2019). Principles and recent applications of novel non-thermal processing technologies for the fish industry—A review. Crit. Rev. Food Sci. Nutr..

[b1170] Zhou D., Wang Z., Tu S., Chen S., Peng J., Tu K. (2019). Effects of cold plasma, UV-C or aqueous ozone treatment on *Botrytis cinerea* and their potential application in preserving blueberry. J. Appl. Microbiol..

[b1175] Zhou R., Zhou R., Prasad K., Fang Z., Speight R., Bazaka K., Ostrikov K.K. (2018). Cold atmospheric plasma activated water as a prospective disinfectant: the crucial role of peroxynitrite. Green Chem..

[b1180] Ziuzina D., Misra N.N., Han L., Cullen P.J., Moiseev T., Mosnier J.P., Bourke P. (2020). Investigation of a large gap cold plasma reactor for continuous in-package decontamination of fresh strawberries and spinach. Innovative Food Sci. Emerg. Technol..

[b1185] Ziuzina D., Patil S., Cullen P.J., Keener K.M., Bourke P. (2014). Atmospheric cold plasma inactivation of *Escherichia coli, Salmonella enterica* serovar *Typhimurium* and *Listeria monocytogenes* inoculated on fresh produce. Food Microbiol..

